# The Apaf-1 apoptosome induces formation of caspase-9 homo- and heterodimers with distinct activities

**DOI:** 10.1038/ncomms13565

**Published:** 2016-11-24

**Authors:** Chu-Chiao Wu, Sunhee Lee, Srinivas Malladi, Miao-Der Chen, Nicholas J. Mastrandrea, Zhiwen Zhang, Shawn B. Bratton

**Affiliations:** 1 Department of Epigenetics and Molecular Carcinogenesis, The University of Texas MD Anderson Cancer Center, Smithville, Texas 78957, USA; 2 Institute for Cellular and Molecular Biology, The University of Texas at Austin, Austin, Texas 78712, USA; 3 Division of Pharmacology and Toxicology, College of Pharmacy, The University of Texas at Austin, Austin, Texas 78712, USA; 4 Bioengineering Department, Santa Clara University, Santa Clara, California 95053, USA

## Abstract

According to dogma, initiator caspases are activated through proximity-induced homodimerization, but some studies infer that during apoptosis caspase-9 may instead form a holoenzyme with the Apaf-1 apoptosome. Using several biochemical approaches, including a novel site-specific crosslinking technique, we provide the first direct evidence that procaspase-9 homodimerizes within the apoptosome, markedly increasing its avidity for the complex and inducing selective intramolecular cleavage at Asp-315. Remarkably, however, procaspase-9 could also bind via its small subunit to the NOD domain in Apaf-1, resulting in the formation of a heterodimer that more efficiently activated procaspase-3. Following cleavage, the intersubunit linker (and associated conformational changes) in caspase-9-p35/p12 inhibited its ability to form homo- and heterodimers, but feedback cleavage by caspase-3 at Asp-330 removed the linker entirely and partially restored activity to caspase-9-p35/p10. Thus, the apoptosome mediates the formation of caspase-9 homo- and heterodimers, both of which are impacted by cleavage and contribute to its overall function.

## Abstract (web summary)

Apoptotic initiator caspases are thought to be activated through homodimerization but this remains controversial. Here the authors demonstrate that caspase-9 can adopt two distinct conformations within the Apaf-1 apoptosome, each with distinct properties that contribute to the overall function of the complex.

## Graphical abstract

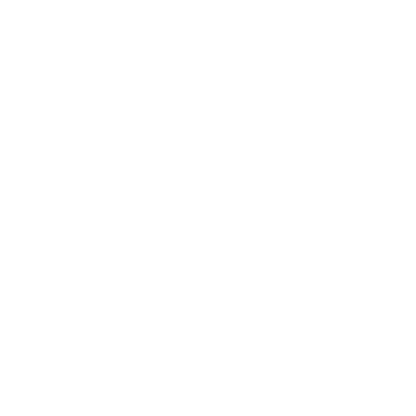

During apoptosis, monomeric ‘initiator' caspases (cysteine proteases), possessing long prodomains, such as caspases-8, -9 and -10, are recruited to and activated within large caspase-activating complexes composed of specific adapter proteins^1^. These apical proteases in turn activate ‘effector' caspases-3, -6 and -7, present in the cell as constitutive dimers, through cleavage of the intersubunit linkers connecting their large and small subunits. Once activated, these downstream caspases dismantle the cell through targeted cleavage of hundreds of substrates^1^,^2^. In the so-called ‘intrinsic' apoptosis pathway, various forms of cellular stress activate proapoptotic BCL-2 family members and stimulate mitochondrial outer membrane permeabilization (MOMP), resulting in the release of cytochrome *c* (Cc) from the intermembrane space into the cytoplasm^3^. Cc, in combination with (d)ATP, then stimulates oligomerization of apoptosis protease-activating factor 1 (Apaf-1) into an ‘apoptosome' complex that recruits caspase-9 (C9) via caspase recruitment domains (CARDs) present in both proteins^4^,^5^.

All initiator caspases are thought to be activated within their caspase-activating complexes through ‘proximity-induced homodimerization'^6^, and indeed, the crystal structures of several initiator caspases reveal apparent weak dimerization motifs near the C termini of their small subunits that, when mutated, inactivate the proteases^1^,^6^. Moreover, enforced dimerization of these caspases, either through fusion with strong dimerization domains or incubation with kosmotropic salts, generally leads to their activation^6^,^7^,^8^,^9^. However, for technical reasons, to date, there has been no direct biochemical proof that initiator caspases dimerize within their respective caspase-activating complexes, and at least in the case of C9, the activities observed following enforced dimerization are reportedly less robust than those resulting from fully reconstituted Apaf-1-C9 apoptosome complexes^8^,^10^. Thus, a competing holoenzyme model has emerged, wherein oligomerized Apaf-1 is proposed to induce allosteric conformational changes in monomeric C9 that result in its activation^10^,^11^,^12^,^13^.

Importantly, C9 must bind and remain bound to the apoptosome to exhibit significant catalytic activity at physiological concentrations, and unlike caspase-8 (C8), its prodomain is not removed following its activation^13^,^14^,^15^,^16^. Moreover, procaspase-9 (ProC9) does not require autocatalytic cleavage of the intersubunit linker for its activation^14^,^15^,^17^,^18^. In fact, we have previously discovered that ProC9 possesses higher affinity for the apoptosome, compared with cleaved C9-p35/p12, and that this difference in affinity facilitates a continuous cycle of ProC9 recruitment/activation, autocatalytic cleavage, and release from the complex^14^. Since autoprocessing is rapid, cleaved C9 is primarily responsible for the downstream activation of procaspase-3 (ProC3) but also slowly dissociates from the complex. Thus, in our view, the purpose of ProC9 autoprocessing is not to activate C9 *per se* but rather to initiate a ‘molecular timer' that regulates the duration of apoptosome activity^14^. In the present study, using a combination of novel biochemical approaches, we demonstrate that both proximity-induced homodimerization and allosteric regulation of monomeric C9 play unique and essential roles in apoptosome function and are integrated well within the overall model of the apoptosome as a molecular timer.

## Results

### ProC9 autoprocessing at Asp-315 inhibits homodimerization

**Figure 1:**
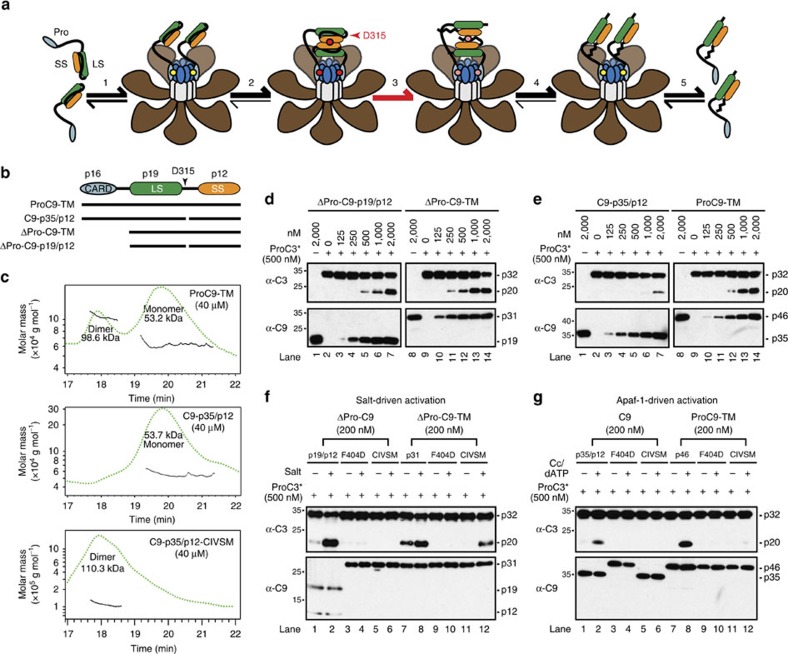
C9 processing at Asp-315 inhibits homodimerization and ProC3 cleavage. (**a**) Model to explain why processing of ProC9 might lead to a reduction in the affinity of C9-p35/p12 for the apoptosome. In this model, ProC9 is first recruited to the apoptosome through what is initially a weak single-site interaction (yellow dot), involving an Apaf-1 CARD and a C9 prodomain (step 1); however, two bound ProC9 monomers interact with one another through dimer motifs in their small subunits, resulting in a network of three binding sites (red dots) that, collectively, are much stronger and stabilize the ProC9 homodimer (step 2); autocatalytic cleavage of ProC9 at Asp-315 (that is, activation of the ‘molecular timer') untethers the intersubunit linker at one end, and the linker (crooked line), along with conformational changes associated with cleavage, destabilize the C9-p35/p12 homodimer and weaken the overall network of interactions (pink dots; step 3); the C9-p35/p12 homodimer dissociates, leaving only the weak Apaf-1 CARD:C9 prodomain interactions (step 4); and the processed C9–35/p12 monomers dissociate from the apoptosome (step 5). (**b**) Depiction of non-cleavable ProC9-TM and two-chain C9-p35/p12, as well as their ‘prodomain-less' versions. (**c**) ProC9-TM, C9-p35/p12 and C9-p35/p12-CIVSM (40 μM) were analysed by SEC-MALS, as described in the Methods section. Ultraviolet absorbance (280 nM) tracings are represented by the green-hatched lines, and the black lines refer to the molar masses (*Y* axis) for each eluted monomer or dimer. (**d**,**e**) ΔPro-C9-p19/p12, ΔPro-C9-TM, C9-p35/p12 or ProC9-TM (125 nM-2 μM) were incubated for 30 min at 37 °C with ProC3* (500 nM) and then immunoblotted for ProC3* cleavage. (**f**,**g**) ΔPro-C9-p19/p12, ΔPro-C9-TM, C9-p35/p12 or ProC9-TM, as well as their corresponding F404D and CIVSM mutants (200 nM), were incubated for 60 (**f**) or 30 min (**g**) at 37 °C with ProC3* (500 nM)±ammonium citrate (1 M) or oligomerized Apaf-1 (300 nM), respectively, and immunoblotted for ProC3* cleavage. (Note that all C9 constructs containing the F404D mutation fail to undergo autocleavage during expression in bacteria due to their inability to homodimerize.) All experiments were repeated at least three times with similar results.

Consistent with the aforementioned molecular timer model, apoptosome-bound non-cleavable ProC9 (ProC9-TM, see Supplementary Table 1 for all C9 constructs) cleaves both the peptide substrate LEHD.amc^14^ and catalytically inactive ProC3-C163A (ProC3*) more robustly and at lower enzyme concentrations than fully processed C9-p35/p12 (Supplementary Fig. 1a). Notably, we previously demonstrated that no differences exist in the apparent *K*_m_ values for ProC9-TM and C9-p35/p12; only the *V*_max_ values differed, in accordance with their individual affinities for the apoptosome^14^. It was unclear from our previous studies, however, why processing of ProC9 *per se* triggered release of the resulting C9-p35/p12 from the complex. According to the proximity-induced homodimerization model of initiator caspase activation, once formed, the apoptosome should recruit ProC9, resulting in an increase in its local concentration that enables formation of a ProC9 homodimer (Fig. 1a, steps 1–2; see figure legend for details). In this scenario, the overall avidity of C9 for the apoptosome should theoretically increase, roughly proportional to the mathematical product of its individual affinities^19^,^20^. In other words, as C9 transitions from a bound monomer, which associates with the apoptosome solely through prodomain-Apaf-1 CARD interactions, to a bound dimer that also interacts via its small subunits, its affinity for the complex should increase markedly (Fig. 1a, step 2).

We therefore speculated that irreversible autocatalytic cleavage of the intersubunit linker in ProC9 at Asp-315 might lead to a decrease in the affinity of C9-p35/p12 for itself, thereby destabilizing the C9 homodimer and initiating the release of C9 monomers from the apoptosome (Fig. 1a, steps 3–5). While this was an attractive mechanistic explanation for how the molecular timer was activated, an intense debate has existed within the field for some time, regarding whether C9 is activated within the apoptosome through proximity-induced homodimerization or through allosteric interactions involving its catalytic subunits^6^,^7^,^8^,^10^,^11^,^12^,^13^,^21^. Therefore, to rigorously interrogate our hypothesis, we began by assessing the relative abilities of pure ProC9-TM and C9-p35/p12 to form homodimers. Using size-exclusion chromatography coupled to multi-angle light scattering (SEC-MALS), we found that at a very high concentration (40 μM), recombinant ProC9-TM, but not C9-p35/p12, formed homodimers in solution (Fig. 1b,c). Importantly, the lower affinity of C9-p35/p12 for itself did not result merely from separation of its large and small subunits, as the processed enzyme remained intact at very low concentrations (Supplementary Fig. 1b–d).

Next, to determine if differences in the activities of pro- and processed C9 were due to differences in their abilities to homodimerize, we first incubated (in the absence of Apaf-1) increasing concentrations of recombinant ProC9-TM, C9-p35/p12 or their ‘prodomain-less' versions, with ProC3* (Fig. 1b,d,e). As anticipated, ΔPro-C9-TM and ProC9-TM exhibited significantly greater activities (ProC3* cleavage) at lower enzyme concentrations than their fully cleaved counterparts, ΔPro-C9-p19/p12 and C9-p35/p12 (Fig. 1d,e). Interestingly, while the C9 prodomain is not removed during apoptosis, the fact that its artificial removal enhanced the relative activities of both unprocessed and processed C9 suggests that it likely interferes with C9 homodimerization in the absence of oligomerized Apaf-1 (ref. 6). We next induced homodimerization by artificially incubating ΔPro-C9-p19/p12 or ΔPro-C9-TM with a high concentration of the kosmotropic salt, ammonium citrate (Fig. 1f)^6^, or by reconstituting Apaf-1 apoptosome complexes with full-length versions of each C9 protein (Fig. 1g). ProC9-TM, as expected, exhibited greater activity than C9-p35/p12 within the apoptosome (Fig. 1g; compare lanes 1, 2, 7 and 8)^14^, but interestingly, the prodomain-less versions of each displayed similar levels of activity in the presence of ammonium citrate (Fig. 1f; compare lanes 1, 2, 7 and 8), implying that both the cleaved and uncleaved enzymes were capable of equivalent activities if homodimerization was fully enforced^14^. Finally, the proposed dimer interfaces of all caspases are located within the C termini of their small subunits, and in the case of C9, mutation of the GCFNF dimerization motif is thought to disrupt homodimerization (Supplementary Fig. 1e,f). We therefore introduced an F404D mutation into each of the full-length and prodomain-less versions of pro- and processed C9, and found that each enzyme failed to cleave ProC3* (Fig. 1f,g; top panels, lanes 3, 4, 9 and 10). Thus, overall, our initial experiments in Fig. 1 suggested that ProC9 possessed a higher affinity for itself, compared with processed C9-p35/p12, and that this correlated with an enhanced ability to cleave ProC3.

### C9 homodimers stably associate with the apoptosome

**Figure 2:**
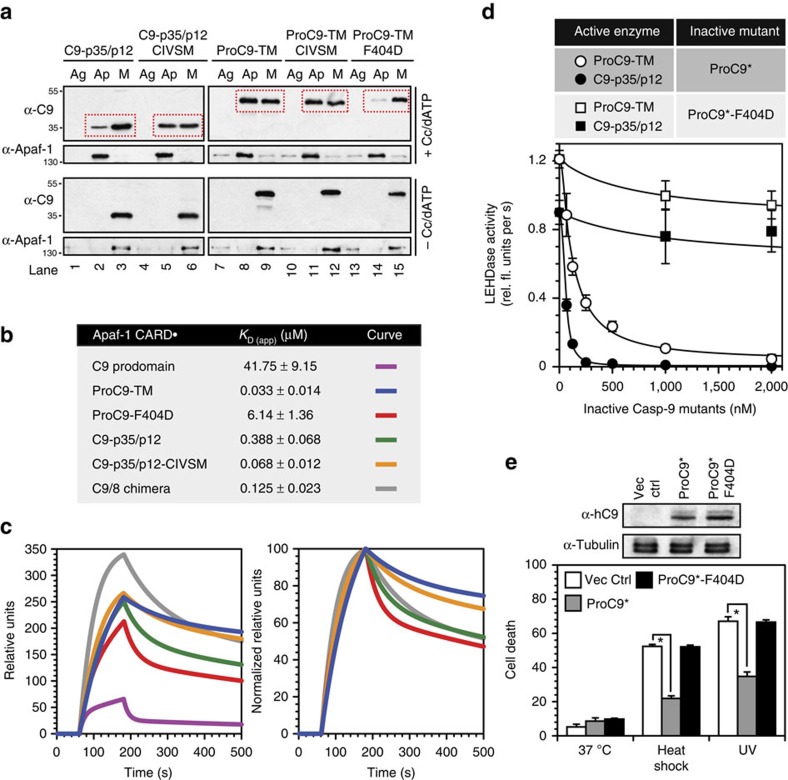
C9 homodimerization stabilizes its association with the apoptosome *in vitro* and in cells. (**a**) Apaf-1 (300 nM) was incubated with C9-p35/12, C9-p35/p12-CIVSM, ProC9-TM, ProC9-TM-CIVSM or ProC9-TM-F404D (200 nM)±Cc/dATP (2 mM each) for 30 min at 37 °C in a final volume of 200 μl. Following separation by Superose-6 gel filtration chromatography, protein fractions corresponding to monomeric proteins (M), apoptosome complexes (Ap) and protein aggregates (Ag) were pooled, precipitated with trichloroacetic acid, separated by SDS–PAGE and immunoblotted for Apaf-1 and C9. The experiments were repeated three times with similar results. (**b**,**c**) Binding affinities of Apaf-1 CARD for C9 prodomain, ProC9-TM, ProC9-TM-F404D, C9-p35/p12, C9-p35/p12-CIVSM and C9/8 were determined by SPR, as described in the Methods section. Analysis of all proteins was repeated five or more times using at least two different streptavidin chips, and each value represents the mean±s.e.m. Left panel: raw curves at 2 μM for each protein; right panel: sensograms for each of the full-length C9 proteins were normalized to their own peak heights to highlight differences in *k*_off_. (**d**) Apoptosome complexes were reconstituted with ProC9-TM or C9-p35/p12 (200 nM)±catalytically inactive ProC9* or ProC9*-F404D (67.5 nM–2 μM) and then assayed for LEHDase activity. Each protein was assayed at least four times, and each value represents the mean±s.e.m. (**e**) Wild-type mouse embryonic fibroblasts (MEFs) expressing either ProC9* or ProC9*-F404D were exposed to heat shock for 1 h or ultraviolet irradiation for 4 min and assayed for cell death at 24 h by propidium iodide (PI) staining/flow cytometry^43^. The experiment was repeated four times, and each bar represents the mean±s.e.m. *P<0.05, analysis of variance, Student–Newman–Keuls *post hoc* analysis.

Although an ability to homodimerize correlated with C9 activity, our experiments did not directly address the impact of C9 processing and/or homodimerization on the association of C9 with the apoptosome. Therefore, to assess the stability of apoptosome-bound ProC9-TM versus C9-p35/p12, we first reconstituted apoptosome complexes with pro- or processed C9, separated the bound from unbound C9 by gel-filtration chromatography and immunoblotted the resulting fractions (Supplementary Fig. 2a,b). Pooled fractions, corresponding to aggregated proteins, of which none were present (Ag), apoptosome-associated proteins (Ap) and monomeric (M) Apaf-1 and C9 were also examined, side by side, in the same gels to better assess the total amounts of bound and unbound C9. As shown in Fig. 2a, in the absence of Cc and dATP, Apaf-1 and C9 proteins eluted as monomers (lower panels, lanes 3 and 9). However, following Cc/dATP-dependent formation of the apoptosome, significantly more ProC9-TM could be found associated with oligomerized Apaf-1, compared with C9-p35/p12 (Fig. 2a; upper panels, compare lanes 2 and 3 with lanes 8 and 9; note the Ap:M ratio for C9 in the hatched red boxes); whereas ProC9-TM (F404D), which could not homodimerize, was incapable of being recruited to the apoptosome (Fig. 2a, lanes 14 and 15, and Supplementary Fig. 2c).

We next questioned whether recruitment of C9-p35/p12 to the apoptosome could be enhanced by enforcing dimerization. We therefore replaced the GCFNF motif in C9 with the dimerization domain in C3 (CIVSM) (Supplementary Fig. 1f), as it reportedly results in a constitutive C9 homodimer^10^. We confirmed its dimerization by SEC-MALS (Fig. 1c), but to our surprise, while full-length ProC9-CIVSM underwent complete processing to C9-p35/p12-CIVSM during expression in bacteria, removal of the prodomain from this construct blocked autocleavage (Fig. 1f,g; lower panels, lanes 5 and 6). Moreover, replacing GCFNF with CIVSM failed to stimulate C9 activity, except when introduced into ΔPro-C9-TM and, even then, only in the presence of high salt (Fig. 1f,g; lanes 5, 6, 11 and 12). Nevertheless, recruitment of C9-p35/p12-CIVSM to the apoptosome was markedly enhanced, compared with the wild-type processed enzyme, and was virtually identical to that of ProC9-TM (Fig. 2a, lanes 2, 3, 5, 6, 8 and 9, and Supplementary Fig. 2d). Thus, while swapping the GCFNF motif for CIVSM inhibited C9 activity, regardless of its processed state, engineering a stable C9-p35/p12 homodimer markedly enhanced its affinity for the apoptosome.

To more precisely determine the impact of C9 processing on the affinities of C9 dimers for the apoptosome, we established a novel binding assay using surface plasmon resonance (SPR), whereby chips, coated with Apaf-1 CARDs, were exposed to various pro- and processed C9 proteins, including dimer interface mutants (Fig. 2b). Consistent with the dimerization, apoptosome recruitment and activity assays already described, we observed that ProC9-TM (*K*_D_=33 nM) bound ∼10-fold more tightly to the Apaf-1 CARDs than did processed C9-p35/p12 (*K*_D_=388 nM), and given that intracellular concentrations of C9 are in the very low nanomolar range^18^, this explains why processing of ProC9 leads to its release from the apoptosome and limits re-recruitment of C9-p35/p12 (Fig. 2a). As would be predicted, introduction of the F404D mutation into ProC9 (*K*_D_=6.1 μM) markedly reduced its affinity for the Apaf-1 CARDs, whereas replacement of the GCFNF motif in C9-p35/p12 with CIVSM (*K*_D_=68 nM) significantly improved its affinity for the Apaf-1 CARDs (Fig. 2b,c). Notably, while the Apaf-1 CARD-C9 prodomain interaction was previously reported to be very strong, capable of withstanding 2 M NaCl (ref. 22), we found this interaction to be relatively weak (*K*_D_=41.8 μM) (Fig. 2b,c). Thus, based on our binding studies, individually, neither the CARD-prodomain nor the C9 homodimer affinities were sufficient to mediate stable formation of the Apaf-1-C9 apoptosome complex. Stability resided in the fact that three individual interactions existed, involving two Apaf-1 CARD-C9 prodomain interactions and the C9 small subunit homodimer, the latter of which was destabilized following cleavage of the intersubunit linker (Fig. 1a).

Finally, to further validate this point, we turned our attention to catalytically inactive ProC9-C287A (ProC9*; Supplementary Table 1), which is an effective dominant-negative inhibitor of cell death initiated by the intrinsic pathway. Its effectiveness results from its ability to compete away the recruitment of endogenous C9 to the apoptosome^14^,^23^. We therefore directly compared ProC9* with ProC9*-F404D, both of which exist in their proforms but only one of which can homodimerize. As predicted, ProC9* suppressed apoptosome activity in reconstitution assays and inhibited heat shock and ultraviolet-induced apoptosis in cells, whereas ProC9*-F404D failed to do so (Fig. 2d,e). Thus, dominant-negative C9 was only effective at inhibiting caspase activation and cell death when it could homodimerize and form a more stable complex with the apoptosome. This also explains why alternative splice forms of C9, which serve as effective endogenous inhibitors of the apoptosome, retain both their prodomains and C-terminal dimerization motifs^24^.

### C9 dimerization is essential for apoptosome activity

**Figure 3:**
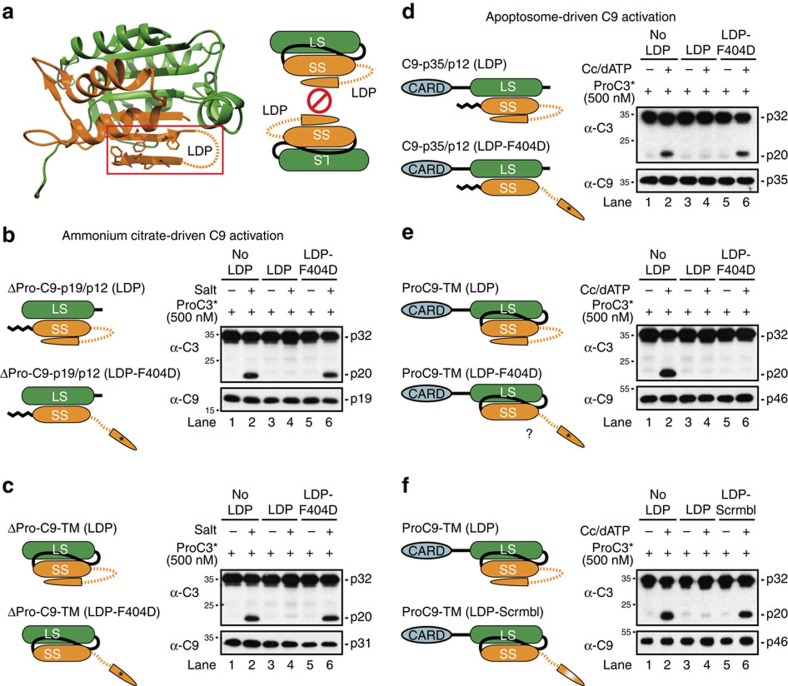
Inhibition of C9 homodimerization with a tethered LDP blocks C9 activity. (**a**) Structural view of C9-LDP fusions: ProC9-TM and C9-p35/p12 were fused with a C-terminal GGSGGSGCFNF. In control constructs, the GCFNF motif in the LDP was mutated (GCDNF), as indicated by an asterisk, or scrambled (FNGFC), as indicated by an orange/white radial colour scheme. (**b**,**c**) ΔPro-C9-p19/p12, ΔPro-C9-p19/p12 (LDP), ΔPro-C9-p19/p12 (LDP-F404D), ΔPro-C9-TM, ΔPro-C9-TM (LDP) or ΔPro-C9-TM (LDP-F404D) proteins (100 nM) were incubated±ammonium citrate (1 M) for 20 min at 37 °C with ProC3* (500 nM). Processing of ProC3* was determined by immunoblotting. (**d**–**f**) Apaf-1 apoptosome complexes were similarly reconstituted with C9-p35/p12, C9-p35/p12 (LDP), C9-p35/p12 (LDP-F404D), ProC9-TM, ProC9-TM (LDP), ProC9-TM (LDP-F404D) or ProC9-TM (LDP-Scrmbl) proteins (100 nM) and incubated for 10 min at 37 °C with ProC3* (500 nM). All experiments were repeated at least three times with similar results.

While the use of dimer interface mutants, such as F404D to disrupt dimerization and GCFNF→CIVSM to promote dimerization, proved extremely useful in assessing the role of dimerization in the recruitment and stabilization of C9 within the apoptosome, mutations within or near this interface in C9 and other caspases impact their catalytic activities and could do so independent of their effects on dimerization (Fig. 1f,g; lanes 3–6 and 9–12)^7^,^25^. Thus, we devised an alternative approach for inhibiting dimerization that did not involve direct mutation of the dimerization motif. On the basis of the C9 crystal structure, the GCFNF motif in one C9 protein binds in an antiparallel manner to the same motif in the adjacent C9 protein (Supplementary Fig. 1e). Therefore, since this dimerization motif is located at the C terminus of C9's small subunit, we generated C9 proteins that contained a C-terminal Gly–Ser linker followed by a GCFNF peptide, which we termed the ‘linker dimer peptide' or LDP (Fig. 3a). We predicted that the tethered GCFNF motif in the LDP would bind to the same motif in the small subunit of C9, in its normal antiparallel manner, and thus inhibit dimerization with another C9 monomer (Fig. 3a).

We initially tested our approach using kosmotropic salt-driven activation of processed and unprocessed C9 proteins, each of which lacked their prodomains but possessed the aforementioned LDPs. Moreover, to verify specificity, we introduced the F404D mutant into the LDP, rather than the small subunit. As predicted, C-terminal fusion of the LDP to ΔPro-C9-TM and ΔPro-C9-p19/p12 markedly inhibited salt-induced activation of C9 and cleavage of ProC3* (Fig. 3b,c; lanes 2 and 4). Moreover, inhibition was specific, as C9 proteins fused to the LDP-F404D readily cleaved ProC3*, exhibiting activities similar to those proteins lacking LDPs altogether (Fig. 3b,c; compare lanes 2 and 6). Our assay further supported the notion that homodimerization was the mechanism for salt-driven C9 activation, but more importantly, given our success, we then utilized the same approach with full-length ProC9-TM and C9-p35/p12 LDP fusion proteins in apoptosome reconstitution assays. As with the salt-driven activations, presence of the LDP completely inhibited C9-p35/p12 activity, which was restored on introduction of the F404D mutation into the LDP (Fig. 3d). Moreover, identical results were observed when lysates from *C9*^*−/−*^ mouse embryonic fibroblasts (MEFs) were incubated with the same recombinant C9 proteins, in the presence of Cc and dATP (Supplementary Fig. 3a).

On the other hand, fusion of either the LDP or the LDP-F404D to ProC9-TM resulted in a complete loss of C9 activity, when reconstituted with pure Apaf-1 apoptosome complexes or Cc/dATP-activated *C9*^*−/−*^ lysates (Fig. 3e and Supplementary Fig. 3b). Since the LDP-F404D had no impact on apoptosome-driven activation of C9-p35/p12 (Fig. 3d and Supplementary Fig. 3a), it appeared unlikely that it interfered with C9 prodomain-Apaf-1 CARD interactions. Instead, given that fusion of the LDP-F404D had little effect on salt-induced activation of ΔPro-C9-TM (Fig. 3c), the most likely explanation was that, under the low salt conditions required for apoptosome formation, the wild-type GCFNF motif in the small subunit of ProC9-TM maintained some affinity for the mutant GCDNF motif in the tethered LDP-F404D (Fig. 3e). To confirm this hypothesis, we generated a ProC9-TM protein, fused to a scrambled LDP (that is, FNGFC), and found that reconstitution of apoptosome complexes with this fusion protein exhibited activity that was indistinguishable from ProC9-TM (Fig. 3f; lanes 2 and 6). Collectively, these experiments strongly suggested that C9 dimerization was essential for its activation within the apoptosome.

### ProC9 forms homo- and heterodimers within the apoptosome

**Figure 4:**
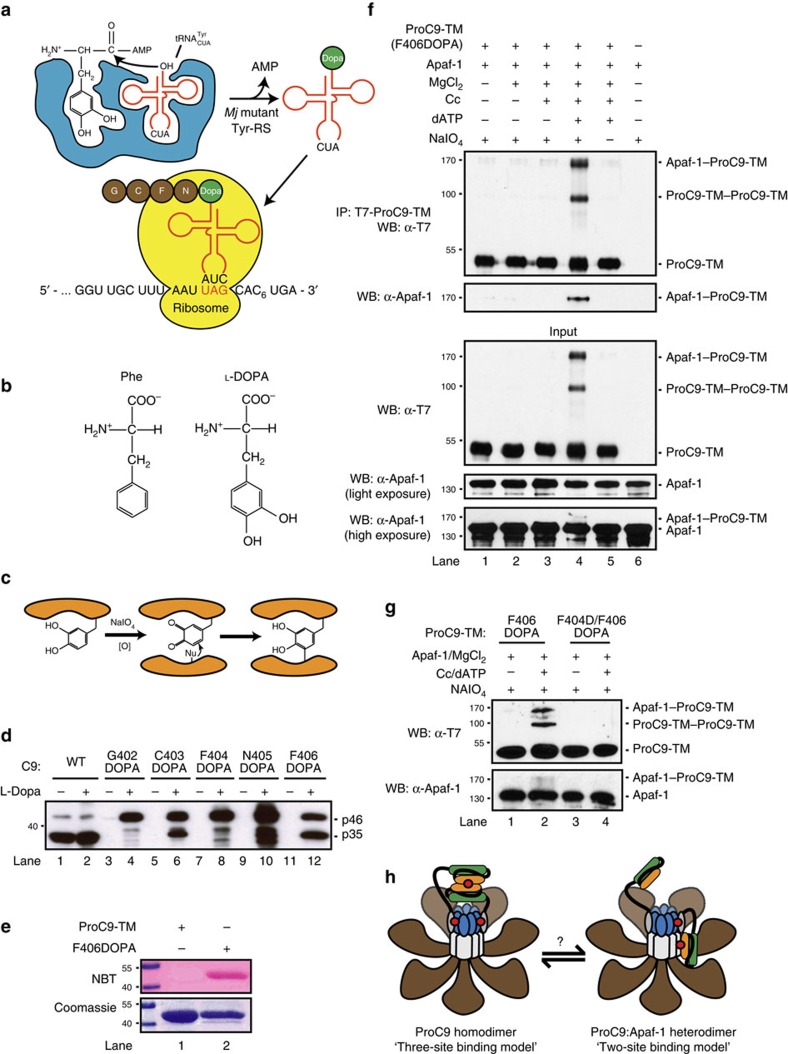
Site-specific incorporation of an unnatural crosslinkable amino acid into ProC9 reveals apoptosome-dependent formation of ProC9 homodimers and ProC9-Apaf-1 heterodimers. (**a**) Model of the engineered *M. jannaschii* tRNA^Tyr^/tyrosyl-tRNA synthetase pair, incorporating L-DOPA into the primary amino acid sequence of ProC9 at an amber stop codon. (**b**,**c**) Structures of Phe and L-DOPA and the proposed mechanism for quinone-mediated protein crosslinking. (**d**) L-DOPA was incorporated into ProC9 at each position in the putative GCFNF dimer motif, as described in the Methods section. (**e**) L-DOPA incorporation into ProC9-TM was confirmed using the nitroblue tetrazolium assay^28^. (**f**,**g**) Apoptosome complexes (300 nM Apaf-1) were reconstituted with either ProC9-TM-F406DOPA or ProC9-TM-F404D/F406DOPA (200 nM), and the incorporated L-DOPA was oxidized with NaIO_4_, generating covalently linked ProC9-TM-ProC9-TM homodimers and ProC9-TM-Apaf-1 heterodimers. All experiments were repeated at least three times with similar results. (**h**) Site-specific crosslinking experiments suggested two distinct binding states or conformations for ProC9 within the Apaf-1 apoptosome, as a ProC9 homodimer and a ProC9-Apaf-1 heterodimer. (Red dots highlight the three-site versus two-site binding models for the homo- and heterodimers, respectively.)

Our affinity and activity assays for pro- and processed C9 homodimers, alone and bound to Apaf-1 CARDs, as well as our use of LDPs, indirectly proved that C9 dimerization was essential for its activity (Figs 1, 2, 3). However, it must be conceded that no initiator caspase, to our knowledge, has ever been directly shown to form a homodimer within its caspase-activating complex. We therefore sought to formally demonstrate that C9 homodimerized within the apoptosome via its GCFNF motif. To overcome the technical difficulties associated with the use of traditional chemical crosslinkers, we instead utilized a novel *Methanococcus jannaschii* tRNA^Tyr^_CUA_/tyrosyl-tRNA synthetase mutant pair to systematically replace each amino acid within the putative C9 dimerization motif with the unnatural amino acid 3,4-dihydroxy-L-phenylalanine (L-DOPA) (Fig. 4a,b and Supplementary Fig. 1e,f)^26^,^27^. Following oxidation of the incorporated L-DOPA with sodium periodate, we predicted that the resulting ortho-quinone would crosslink nucleophilic amino acids in proteins bound to C9 through this region (Fig. 4c). Importantly, these C9 proteins were expressed only when bacteria were grown in the presence of L-DOPA, and judging by the degree of autoprocessing, incorporation of L-DOPA at Phe-406 had the least negative impact on C9 activity (Fig. 4d). This was not entirely surprising given the structural similarity of phenylalanine to L-DOPA (Fig. 4b). Since L-DOPA can undergo redox cycling to generate superoxide, incorporation of L-DOPA into ProC9-TM was also confirmed using the nitroblue tetrazolium assay (Fig. 4e)^28^.

Finally, we performed apoptosome reconstitution assays using recombinant full-length Apaf-1 and ProC9-TM-F406DOPA. Following separation of proteins by SDS–polyacrylamide gel electrophoresis (SDS–PAGE), ProC9-TM-F406DOPA clearly formed crosslinkable homodimers (Fig. 4f; lower panels), but unexpectedly, ProC9-TM-F406DOPA also directly crosslinked Apaf-1, which was further confirmed by immunoprecipitation (Fig. 4f; upper panels). Similar results were obtained when *C9*^*−/−*^ lysates were reconstituted with ProC9-TM-F406DOPA (Supplementary Fig. 4). The crosslinked ProC9-TM homodimers and ProC9-TM-Apaf-1 heterodimers were formed only in the presence of fully assembled apoptosome complexes and following the oxidation of L-DOPA (Fig. 4f and Supplementary Fig. 4). Moreover, both crosslinks were disrupted by incorporation of the F404D mutation into ProC9-TM-F406DOPA (Fig. 4g). By contrast, despite repeated efforts, we were unable to detect clear evidence of homo- or heterodimers with C9-p35/p12-F406DOPA, presumably due to its significantly lower affinity for the apoptosome (data not shown). Given its proximity, we suspect that oxidized DOPA-406 most likely reacted with the sulphur in Cys-403 of the adjacent C9 protein to form a stable thioether bond (Supplementary Fig. 1e, inset). Unfortunately, even a conservative C403S mutation significantly inhibited C9 autoprocessing in bacteria, even before incorporation of the F406DOPA mutation (data not shown). Regardless, the results demonstrated, for the first time, that ProC9 forms homodimers within the apoptosome and, interestingly, that it also binds directly to Apaf-1 via the same dimerization motif. This led us to speculate that ProC9 might possess two active conformations within the apoptosome, as either ProC9 homodimers or ProC9-Apaf-1 heterodimers, and that it might transition between these two conformations (Fig. 4h).

### Monomeric C9 is active within the Apaf-1 apoptosome

**Figure 5:**
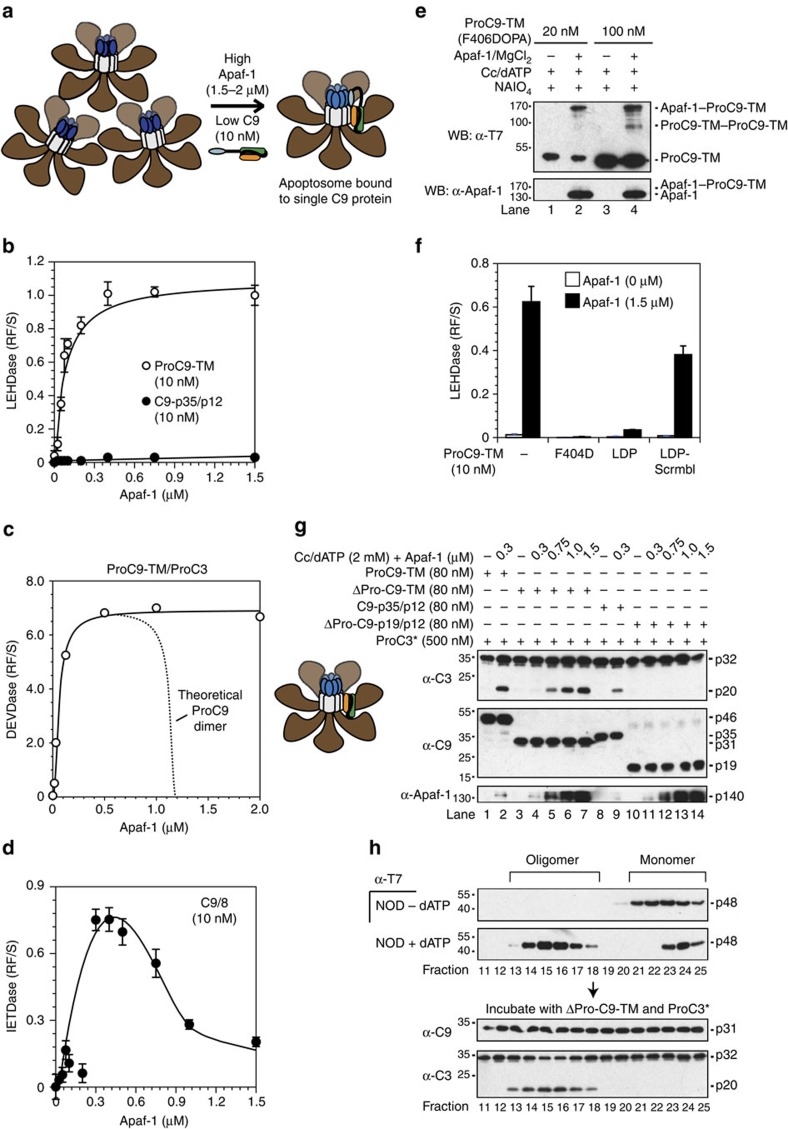
Monomeric ProC9 can be activated through a direct interaction with oligomerized Apaf-1 NODs. (**a**–**d**) In Apaf-1 saturation experiments, ProC9-TM, C9-p35/p12 or C9/8 enzymes (10 nM) were incubated with Cc (10 μM), dATP/MgCl_2_ (2 mM each) and Apaf-1 (25 nM–2 μM), and enzyme activities were directly measured using the C9 or C8 fluorescent substrates, LEHD.amc or IETD.amc, respectively. C9 complexes were also incubated with wild-type ProC3 in a coupled assay that measures C3 DEVDase activity^14^. (**e**) Apoptosome complexes were reconstituted with Apaf-1 (1.5 μM) and ProC9-TM-F406DOPA (20 or 100 nM). The incorporated L-DOPA was then oxidized with NaIO_4_, covalently linking ProC9-TM-ProC9-TM homodimers and/or ProC9-TM-Apaf-1 heterodimers. (**f**) ProC9-TM, ProC9-TM-F404D and the LDP fusions, ProC9-TM (LDP) and ProC9-TM (LDP-Scrmbl) (10 nM) were incubated with or without oligomerized Apaf-1 (1.5 μM) and assayed for LEHDase activity. (**g**) ProC9-TM, ΔPro-C9-TM, C9-p35/p12 and ΔPro-C9-p19/p12 (80 nM) were reconstituted with increasing concentrations of oligomerized full-length Apaf-1 (0.3–1.5 μM) and assayed for cleavage of ProC3* (500 nM). (**h**) Apaf-1 NOD protein (1.5 μM) was incubated±dATP/MgCl_2_ (2 mM each) in a final volume of 200 μl for 30 min at 37 °C. The protein was then fractionated by Superose-6 gel filtration chromatography, and the majority or each fraction (977 μl) was trichloroacetic acid precipitated and the pellet immunoblotted for Apaf-1 NOD. The remaining portion (23 μl) was incubated with ΔPro-C9-TM (200 nM) and ProC3* (500 nM) for 30 min at 37 °C. Samples were then immunoblotted for C9 and C3. With the exception of **c** (*n*=2), all experiments were repeated 3–9 times with similar results, and all data points/bars represent the mean value±s.e.m.

Although the purpose and biological significance of the ProC9-Apaf-1 heterodimer remained unclear, our previous titration experiments with ProC9 hinted that it might be active as a monomer within the apoptosome^14^. Therefore, to isolate the heterodimer, we performed apoptosome saturation experiments, using low concentrations of pro- or processed C9 (10 nM) in combination with increasing amounts of Apaf-1 (25 nM–2.0 μM) (Fig. 5a). We speculated that if C9 could be activated only as a homodimer, then we should observe an ‘inverted-U' dose–response curve, since at high concentrations of Apaf-1 only a single C9 protein should be bound to the apoptosome. Conversely, if C9 could function as an active monomer, then we should observe a saturable dose–response curve. As shown in Fig. 5b, as the concentration of Apaf-1 in our incubations was increased, ProC9-TM LEHDase activity increased markedly, reaching saturation at ∼300–400 nM of Apaf-1, and remained steady at concentrations in excess of 1.5 μM. By contrast, at this low enzyme concentration, processed C9-p35/p12 exhibited detectable but significantly lower activity across the same concentration range of Apaf-1 (Fig. 5b). Furthermore, activation of monomeric ProC9-TM was not merely an artefact of using the peptide substrate LEHD.amc, as identical results were obtained in a coupled reaction, where excess wild-type ProC3 was added to each incubation and C3 DEVDase activity was measured (Fig. 5c)^14^.

Though we felt it unlikely, we considered the possibility that, despite the vast excess of Apaf-1, complexes containing ProC9 homodimers might be selected for. Therefore, to validate our assay, we generated a C9/8 chimeric construct, wherein the prodomain of C9 was fused to the large and small subunits of C8. This construct was previously utilized to demonstrate that the apoptosome could activate C8 through proximity-induced dimerization^7^, and indeed, processed C9/8 possessed relatively high affinity for the Apaf-1 CARDs—significantly higher than processed C9-p35/p12 and near that of ProC9-TM (Fig. 2b). Given that C8 possesses a substantially different dimerization motif than C9 (Supplementary Fig. 1f), it would be highly unlikely for it to directly associate with the apoptosome via its small subunit. Thus, when incubated with increasing concentrations of Apaf-1, the C9/8 chimera exhibited an ‘inverted-U' dose–response curve that would be expected for an initiator caspase that strictly relies on homodimerization for its activation (Fig. 5d). In short, our activity assay could distinguish between active caspase monomers and dimers.

Finally, to further confirm the existence and activity of ProC9-TM-Apaf-1 heterodimers, we performed crosslinking experiments and activity assays in the presence of 1.5 μM Apaf-1. As expected, at 20 nM, ProC9-TM-F406DOPA exclusively crosslinked Apaf-1, failing to produce any detectable homodimers (Fig. 5e; lanes 1 and 2). Mutation of the dimerization motif (F404D) in ProC9-TM also completely blocked apoptosome-dependent LEHDase activity, as did fusion of the LDP (but not the scrambled LDP) to the C terminus of ProC9-TM (Fig. 5f). Thus, together, our data strongly suggested that the Apaf-1 apoptosome could directly activate monomeric C9 through an interaction with its dimerization motif. Notably, however, when we raised the concentration of ProC9-TM-F406DOPA from 20 to 100 nM in our crosslinking experiments, lowering the Apaf-1:ProC9-TM molar ratio from 75:1 to 15:1 (or heptameric apoptosome:ProC9-TM ratio from ∼10:1 to 2:1), we began to observe both crosslinked ProC9-TM homodimers and ProC9-TM-Apaf-1 heterodimers (Fig. 5e; lanes 3 and 4). Therefore, given that C9 concentrations are generally thought to exceed those of Apaf-1 in most cells, C9 is almost certainly present within the apoptosome as both active homo- and heterodimers.

### The Apaf-1 NOD stimulates ProC9-dependent cleavage of ProC3

Our next goals were to identify the region in Apaf-1 responsible for activating C9 and to determine whether C9-Apaf-1 heterodimers were more or less effective than C9 homodimers at cleaving ProC3. As already noted, the high affinity of ProC9 for the apoptosome resulted in large part from its ability to form a network of interactions, involving the homodimer and two independent prodomain-Apaf-1 CARD interactions (Fig. 1a, step 2, and Fig. 2). Nevertheless, even if one prevents proximity-induced homodimerization of C9 by removing its prodomain, ΔPro-C9-TM should still interact with Apaf-1 via its dimer motif, so long as the Apaf-1 concentration is sufficiently high to promote C9 single-site binding. We therefore incubated a fixed concentration of ΔPro-C9-TM or processed ΔPro-C9-p19/p12 with increasing concentrations of reconstituted apoptosome complexes and found that full-length Apaf-1 dose-dependently stimulated ΔPro-C9-TM activity (ProC3* cleavage) far more efficiently than processed ΔPro-C9-p19/p12 (Fig. 5g; top panel, lanes 3–7 and 10–14).

Given structural considerations^29^, we suspected that monomeric ΔPro-C9-TM most likely bound directly to the Apaf-1 nucleotide-binding oligomerization domain (NOD; aa 127–530), as has been proposed for processed C9 in a cryo-electron microscopy model of the apoptosome^11^. Consequently, we expressed and purified Apaf-1 NOD protein, incubated it with ΔPro-C9-TM, in the presence or absence of dATP/MgCl_2_, and subjected the mixture to Superose-6 gel filtration chromatography. As expected, stimulation of Apaf-1 NOD proteins with dATP/MgCl_2_ resulted in the formation of NOD apoptosome complexes that effectively recruited ΔPro-C9-TM (Supplementary Fig. 5a). In a related experiment, we stimulated formation of Apaf-1 NOD complexes (in the absence of ΔPro-C9-TM) and then, following separation by gel filtration, incubated each fraction with ΔPro-C9-TM and ProC3* (Fig. 5h). Only those fractions that contained fully oligomerized NOD proteins stimulated ΔPro-C9-TM-dependent cleavage of ProC3* (Fig. 5h, fractions 13–18). Consistent with this finding, an Apaf-1 NOD p-loop mutant (K160R), which is partially resistant to oligomerization^30^, was also less efficient at stimulating ΔPro-C9-TM activity (Supplementary Fig. 5b; lanes 2–7). The fact that monomeric Apaf-1 NOD (K160R), at high concentrations, weakly stimulated ΔPro-C9-TM activity may suggest that ProC9 makes contact with a single Apaf-1 NOD in the oligomerized complex but requires interactions with an adjacent NOD to stabilize the interaction. (Note that the final Apaf-1 NOD concentrations in Supplementary Fig. 5b were significantly higher than those present in the diluted gel filtration fractions from Fig. 5h.) Regardless, monomeric ProC9 could be activated through a direct interaction with the so-called ‘hub' of the Apaf-1 apoptosome, much more effectively than autocatalytically processed C9-p35/p12.

**Figure 6:**
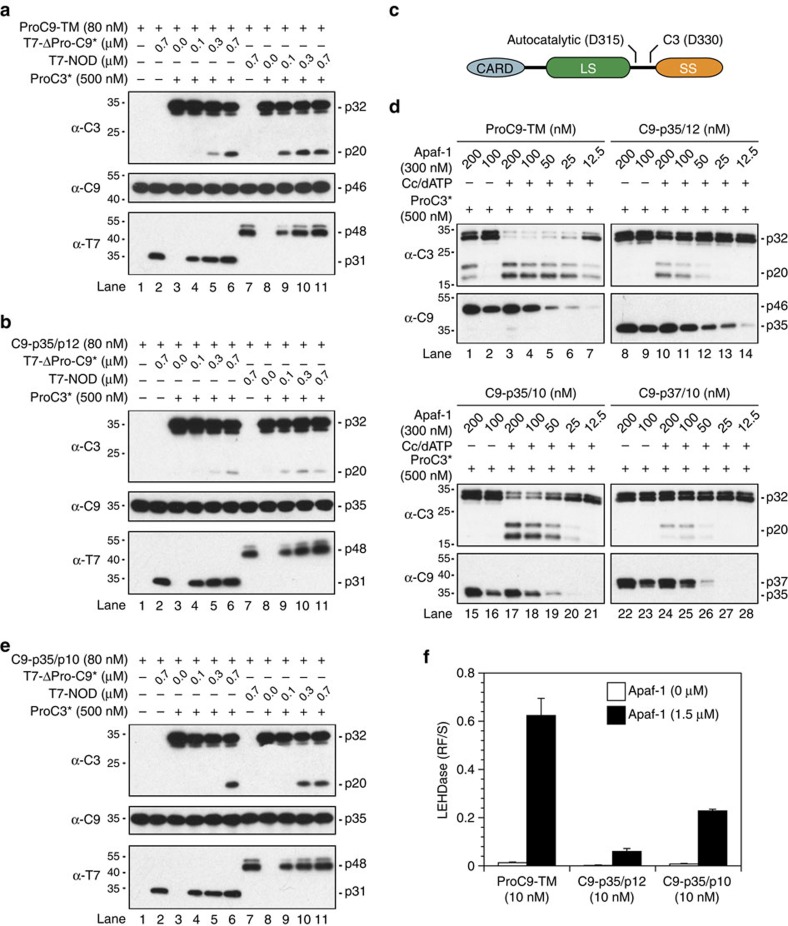
Apaf1 NOD-bound ProC9-TM stimulates ProC3 cleavage more efficiently than C9-p35/p12, but feedback cleavage by active C3 partially restores C9-p35/p10 activity. (**a**,**b**,**e**) ProC9-TM, C9-p35/p12 and C9-p35/p10 (80 nM) were incubated, in the presence of ammonium citrate (1 M), with either T7-tagged ΔPro-C9* or NOD protein (0.1–0.7 μM)±ProC3* (500 nM) for 15 min at 37 °C. Processing of ProC3* was determined by immunoblotting, and enhanced chemiluminescence (ECL) was performed simultaneously on all nitrocellulose membranes. (**c**,**d**) ProC9 undergoes autocatalytic processing at Asp-315 and/or C3-mediated cleavage at Asp-330. Apoptosome complexes were therefore assembled in the presence of ProC9-TM, C9-p35/p12, C9-p35/p10 or C9-p37/p10 (12.5–200 nM). As before, their individual abilities to process ProC3* (500 nM) were determined by western blotting. (**f**) ProC9-TM, C9-p35/p12 and C9-p35/p10 (10 nM) were incubated with a saturating concentration of oligomerized Apaf-1 (1.5 μM), and the Apaf-1-bound monomeric enzymes assayed for LEHDase activity. All experiments were repeated at least three times with similar results, and each bar in **f** represents the mean±s.e.m.

Since both pure Apaf-1-free C9 homodimers and C9-Apaf-1 heterodimers were catalytically active (Figs 1 and 5), it was unclear which interaction resulted in the more efficient cleavage of ProC3. To address this question, we set up assays in which a fixed concentration of full-length ProC9-TM or processed C9-p35/p12 was incubated, in the presence of ammonium citrate (1 M), with increasing concentrations (0.1–0.7 μM) of either catalytically inactive ΔPro-C9-C287A (ΔPro-C9*) (Fig. 6a,b; lanes 3–6) or dATP-activated NOD protein (Fig. 6a,b; lanes 8–11). We used ΔPro-C9* to induce formation of C9 homodimers for two reasons: first, to perform titrations with catalytically active C9 would simply increase the overall amount of activatable enzyme and unfairly bias comparisons with the Apaf-1 NOD. Second, a previous crystal structure of the C9 homodimer revealed that only one of two potential active sites was bound by an inhibitor (Supplementary Fig. 1e)^21^, implying that an ‘inactive' C9 monomer could activate its binding partner through homodimerization. As anticipated, under both conditions, ProC9-TM was more active than processed C9-p35/p12 (Fig. 6a,b). The presence of ammonium citrate did increase NOD-dependent activation of C9-p35/p12, but the activity paled in comparison with ProC9-TM (Fig. 6a,b; lanes 8–11). Equally important, however, Apaf-1 NOD (at lower protein concentrations) stimulated significantly more C9-dependent cleavage of ProC3* than did ΔPro-C9* (Fig. 6a,b; compare lanes 3–6 with lanes 8–11), implying that the Apaf-1-ProC9 heterodimer was more effective at cleaving ProC3 than was the ProC9 homodimer.

### C3 cleavage of C9-p35/p12 restores activity to C9-p35/p10

The fact that cleaved C9-p35/p12 possessed lower affinity for itself and the Apaf-1 NOD, compared with ProC9-TM (Figs 1, 2, 5 and 6), was consistent with our molecular timer model for the apoptosome, wherein cleavage of ProC9 at Asp-315 initiated the release of C9-p35/p12 from the complex^14^. As to why an overall loss in affinity occurred following processing, we hypothesized that the cleaved intersubunit linker might interfere with the dimerization motif in C9 (Fig. 1a, step 3). If true, it was notable that C9 possesses a second cleavage site at Asp-330, which can only be cleaved by effector C3 through a feedback pathway (Fig. 6c)^31^. Since cleavage of C9-p35/p12 at Asp-330 would naturally remove the entire intersubunit linker, we reconstituted apoptosome complexes with increasing concentrations of ProC9-TM, C9-p35/p12, C9-p35/p10 or the C3-derived cleavage product of ProC9, C9-p37/p10. Untethering of the intersubunit linker at either end, following cleavage at Asp-315 or Asp-330, resulted in a marked decrease in apoptosome activity. Indeed, both C9-p35/p12 and C9-p37/p10 cleaved far less ProC3* than did ProC9-TM, particularly at lower, more physiologically relevant enzyme concentrations (Fig. 6d, note both pro- and processed forms of C3). Conversely, C9-p35/p10, generated following complete removal of the intersubunit linker, significantly enhanced C9 activity (Fig. 6d).

We assumed that removal of the linker would impact the ability of C9-p35/p10 to both homodimerize and heterodimerize with Apaf-1 NODs, and indeed, when compared with C9-p35/p12, the activity of C9-p35/p10 was significantly enhanced through direct binding of the monomeric enzyme to excess ΔPro-C9*, oligomerized NODs and Apaf-1 apoptosome complexes (Fig. 6b,e,f). Moreover, catalytically inactive, processed C9-p35/p10* was a better dominant-negative inhibitor of the apoptosome than either C9-p35/p12* or C9-p37/p10* (Supplementary Fig. 6). ProC9 was still more active than C9-p35/p10 (and ProC9* was a better dominant-negative inhibitor), but this was expected given that ProC9 possessed an overall higher affinity for the apoptosome (Fig. 6 and Supplementary Fig. 6). In fact, this difference in affinity is a requisite feature of the molecular timer, as it allows for the continuous recruitment of ProC9, autoprocessing and release from the complex. Nevertheless, proteolytic feedback of C3 on C9-p35/p12 incorporates an elegant ‘threshold' feature into the timer. As active C3 accumulates, it will remove the intersubunit linker from C9 and further increase/restore apoptosome activity, propagating a feed-forward amplification loop that ensures cell death.

### ProC9 homodimerization induces intramolecular cleavage

**Figure 7:**
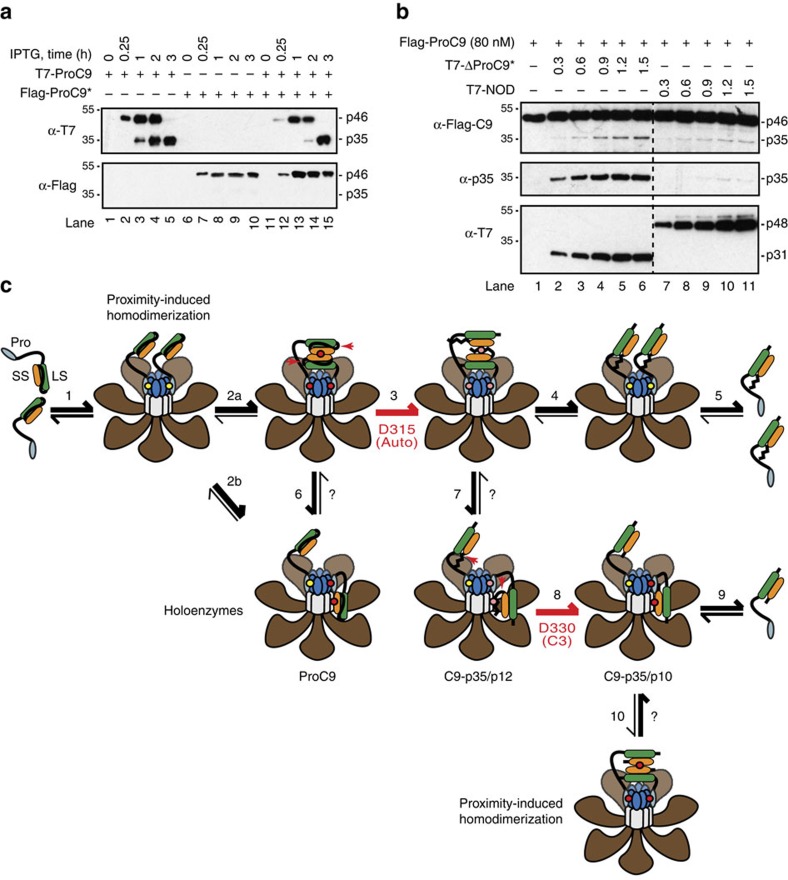
ProC9 undergoes selective intramolecular cleavage following homodimerization. (**a**) T7-tagged ProC9 and Flag-tagged ProC9* were co-expressed in bacteria for 0–3 h, and bacterial lysates were immunoblotted using anti-T7 and Flag antibodies. (**b**) Wild-type Flag-tagged ProC9 (80 nM) was incubated with either T7-ΔPro-C9* or T7-NOD (0.3–1.5 μM) for 30 min at 37 °C. Autocatalytic processing of ProC9 at Asp-315 was then determined by immunoblotting with either an anti-Flag antibody or a p35-specific C9 antibody. All blots are from single gels; the hatched line represents a cropped-out lane. Experiments in **a** and **b** were repeated three times with similar results. (**c**) Consolidation of proximity-induced and holoenzyme models of C9 activation. ProC9 is first recruited to the apoptosome through what is initially a weak single-site interaction (yellow dot), involving an Apaf-1 CARD and a C9 prodomain (step 1); however, once bound, two ProC9 monomers can interact with one another through dimer motifs in their small subunits, resulting in a network of three binding sites (red dots) that collectively stabilize the ProC9 homodimer (step 2a), or a single ProC9 protein can interact directly with the NOD hub of the apoptosome via the same dimerization motif (step 2b). Steps 3–5 are described in the legend to Fig. 1a. Steps 6 and 7 indicate that ProC9 and C9-p35/p12 can adopt either a homo- or heterodimeric conformation, and these conformations may be in equilibrium. Step 8 signifies the irreversible cleavage (red arrow) of C9-p35/p12 at Asp-330 to form C9-p35/p10; this cleavage event could occur when C9-p35/p12 is present as either a homo- or heterodimer, but only the latter is shown for simplicity. As before, C9-p35/p10 can either dissociate from the apoptosome (step 9) or possibly transition between a homodimer and a heterodimer (step 10).

ProC9 is recruited to the apoptosome, where it forms a stable homodimer (Fig. 2); however, both ProC9 and processed C9, particularly C9-p35/p10, interact with the Apaf-1 NOD and appear to exhibit maximal activity as heterodimers (Figs 5 and 6). Since the interaction of C9 homodimers with the apoptosome would involve a three-site binding model, whereas a C9-Apaf-1 heterodimer would require only a two-site binding model (Fig. 4h), this led us to question why the former would even exist. However, we recalled from our previous work that active apoptosome-bound C9 failed to cleave catalytically inactive (bound or unbound) ProC9* in reconstitution assays^14^; and even when co-expressed at high concentrations in bacteria, catalytically active wild-type ProC9 readily underwent autoprocessing but failed to cleave inactive ProC9* (Fig. 7a).

These data indicated that active ProC9 dimers did not cleave one another within or outside the apoptosome through intermolecular processing, but rather that homodimerization induced intramolecular cleavage, that is, that an active ProC9 protein cleaved its own intersubunit linker at Asp-315. To determine whether intramolecular cleavage occurred only as a result of homodimerization, or whether binding of ProC9 to the Apaf-1 NOD could likewise induce this type of cleavage, we purified wild-type ProC9 and, as before, incubated it with ΔPro-C9* or activated Apaf-1 NOD protein (0.3–1.5 μM). Remarkably, in both the absence and presence of ammonium citrate, autocatalytic cleavage of ProC9 occurred almost exclusively in response to homodimerization with ΔPro-C9* and not as a result of binding to NOD apoptosome complexes (Fig. 7b and Supplementary Fig. 7), even though the latter was more efficient at inducing C9-mediated cleavage of ProC3* (Figs 5 and 6). Thus, ProC9 undergoes intramolecular cleavage at Asp-315, selectively following homodimerization.

## Discussion

The initial purpose of this study was to further characterize the Apaf-1 apoptosome as a proteolytic-based molecular timer^14^. In particular, we sought to determine why autoprocessing of ProC9 led to the release of C9-p35/p12 from the apoptosome. In doing so, we developed and utilized a number of novel techniques to formally demonstrate, for the first time, that ProC9 is recruited to the apoptosome through a network of interactions, involving the formation of a ProC9 homodimer and two independent prodomain-Apaf-1 CARD interactions (Figs 1, 2, 3, 4). Intramolecular cleavage of ProC9, induced selectively by homodimerization, then freed the intersubunit linker at one end, and the untethered linker (and conformational changes associated with cleavage) disrupted the resulting C9-p35/p12 homodimer, initiating its release from the apoptosome (Fig. 7b,c, steps 1–5). Even more remarkably, however, our site-specific crosslinking experiments revealed that ProC9 could also bind directly to Apaf-1 via the same dimerization motif (Fig. 4), and Apaf-1 saturation experiments provided strong biochemical evidence that the apoptosome could activate monomeric ProC9. Subsequent deletion and comparative binding/activity assays then demonstrated that the NOD in Apaf-1 could heterodimerize with and stimulate C9-dependent cleavage of ProC3 more efficiently than C9 homodimers (Figs 5 and 6 and Fig. 7c, steps 2b, 6 and 7). The fact that C9 autocleavage occurred predominantly as a result of homodimerization, whereas NOD-bound C9 preferentially activated ProC3, suggests that C9 assumes different conformations as homo- and heterodimers. However, the Apaf-1 NOD may also possess a higher affinity for C9 (than does C9 for itself) and/or contain a docking site for ProC3, all of which are currently under further investigation.

While it remains formally possible that C9 homo- and heterodimers might exist in separate apoptosome complexes, we currently favour a model wherein C9 transitions between these two conformations, in part because having two binding sites for the small subunit of C9, should further increase its affinity for the apoptosome. Moreover, X-linked inhibitor of apoptosis (XIAP) selectively inhibits C9-p35/p12, and since it prevents most apoptosome-dependent cleavage of ProC3 (refs 14, 17, 32, 33, 34), this strongly suggests that the majority of ProC9 is first recruited to the apoptosome as a homodimer, where it undergoes rapid autocatalytic processing. A small fraction of ProC9 may directly heterodimerize with Apaf-1 (Fig. 7, step 2b), which may explain why ProC9 can activate some ProC3, even in the presence of excess XIAP^14^. Regardless, once processed, we speculate that C9-p35/p12 transitions between the homo- and more active heterodimer until it dissociates from the apoptosome. It may seem paradoxical that C9-p35/p12 contributes more to the cleavage of ProC3 than ProC9 when ProC9-TM exhibits greater activity following homo- and heterodimerization (Figs 5 and 6). However, this is precisely the point of the molecular timer. While both ProC9-TM and C9-p35/p12 maintain their associations with the apoptosome and exhibit virtually identical activities at high enzyme concentrations, at more physiologically relevant concentrations, rapid ProC9 autoprocessing activates the molecular timer, and the rate at which C9-p35/p12 dissociates from the complex (and thus loses its capacity to activate ProC3) dictates how fast the timer ‘ticks over'^14^. Given the underlying cause of C9 dissociation from the apoptosome, anything that inhibits or enhances dimerization should correspondingly shorten or prolong the time it takes for the timer to tick over. In this regard, it is worth noting that XIAP binds to the N terminus of the small p12 subunit of C9-p35/p12 and prevents its dimerization^17^,^32^,^35^; whereas feedback cleavage of C9-p35/p12 by active C3 not only removes the necessary binding site for XIAP^32^ but also partially restores the inherent ability of C9-p35/p10 to engage the Apaf-1 NOD (Fig. 6). Thus, removal of the intersubunit linker establishes a feed-forward amplification loop that ensures C9-p35/p10 activates even more ProC3 and enhances cell death (Fig. 7, steps 8–10).

Finally, since our data suggest that Apaf-1 NOD-C9 heterodimers cleave ProC3 more efficiently than C9 homodimers, we speculate that high- and low-activity states may serve biologically important functions within the cell. Caspase activation is required for the differentiation of various cell types, and this often requires C9 and C3 (or their homologues)^36^,^37^. Similarly, recent evidence suggests that, in response to stress, some cells fail to die by apoptosis following limited MOMP. This ‘minority MOMP' instead leads to cellular transformation by inducing caspase-dependent DNA damage and genomic instability^38^,^39^. So how does a cell activate sufficient caspases to promote differentiation, or worse, support transformation, rather than kill the cell? Intriguingly, several proposed regulators of the apoptosome^4^, some of which are associated with cancer, may bind to the Apaf-1 NOD to prevent formation of the more highly active heterodimers and instead may promote more modest activation of ProC3 by C9 homodimers. Intracellular concentrations of ProC9 also directly impact the duration of the apoptosome's molecular timer^14^, and our current study suggests that the relative concentrations of Apaf-1 and ProC9 can impact the proportion of C9 homo- and heterodimers formed within the apoptosome. The extent to which the molecular timer and the relative concentrations of Apaf-1 and C9 impact cell death, differentiation or tumorigenesis *in vivo* is currently unclear but should be testable in the future using genetically engineered mouse models.

## Methods

### Reagents

Bovine Cc (catalogue# C3131) and dATP (catalogue# D4788) were purchased from Sigma. Anti-T7 (catalogue# 69522-3), Flag (catalogue# F7425), Apaf-1 (catalogue# 8969), C3 (catalogue# 9662) and four C9 antibodies (full-length, catalogue# 9502S; p35 cleavage-specific, catalogue# 9505; CARD-specific, catalogue# BDB551246; and small subunit-specific, catalogue# AB6080) were obtained from Novagen, Sigma, Cell Signaling Technologies, BD Biosciences or Abcam, and were utilized at 1:1,000 dilutions for western blotting. LEHD.amc (catalogue# 03AMC154) and DEVD.amc (catalogue# 03AMC138) were acquired from MP Biomedicals, and L-DOPA and Biacore sensor streptavidin chips (catalogue# BR-1000-32) were obtained from Acros and GE Biosciences, respectively.

### Molecular cloning

Full-length C9 and C3 constructs were previously described^14^, and single and compound mutants were generated by site-directed mutagenesis (Supplementary Table 1). C9 constructs, lacking their prodomains (Δ1–137), were cloned into the BamHI/NotI sites of pET28b (Novagen). Apaf-1 CARD, containing a C-terminal GCN4 leucine zipper (RMKQLEDKVEELLSKNYHLENEVARLKKLVGER), followed sequentially by an AviTag (GLNDIFEAQKIEWHE) and a His_6_-tag, was cloned into the *NcoI*/*XhoI* sites of pRSF-Duet1 (Novagen).

### Protein expression, purification and modification

Recombinant full-length Apaf-1XL protein was expressed in Hi-five (Invitrogen) or Sf21 cells using a baculoviral expression system^14^. All recombinant C9, C3, Apaf-1 NOD (127–530), Apaf-1 CARD (1–137) and BirA proteins were expressed in *Escherichia coli* strain BL21(DE3)pLysS (Novagen), following isopropyl-β-D-thiogalactoside induction and incubation overnight at 16–18 °C, or for 3 h at 25–30 °C. To obtain full-length wild-type ProC9 and ProC3, very short (5–10 min) inductions at 25 °C were required. In nature, ProC9 undergoes autoprocessing at PEPD^315^↓A within the apoptosome to generate the two-chain C9-p35/p12 enzyme, and both ProC9 and C9-p35/p12 can be cleaved by active C3 at DQLD^330^↓A to generate C9-p37/p10 and C9-p35/p10, respectively (Supplementary Fig. 8a). Therefore, to generate recombinant C9-p35/p12, wild-type ProC9 was simply overexpressed in bacteria, which induced homodimerization and intramolecular cleavage at Asp-315. Wild-type C9-p35/p10 was produced by substituting the DQLD sequence with PEPD, so that the entire linker (including the newly inserted PEPD sequence) would be autocatalytically removed during overexpression (Supplementary Fig. 8a; left flow chart). Similarly, to generate recombinant C9-p37/p10, the PEPD and DQLD motifs were swapped for one another, so that cleavage would only occur at PEPD^330^↓A (Supplementary Fig. 8a, right flow chart). To generate C9-p35/p12*, C9-p35/p10* and C9–37/p10* proteins, we utilized a similar approach, except that cleaved forms were generated by incubating the catalytically inactive C9 proteins with untagged active C3, which selectively cleaves C9 at DQLD↓A (Supplementary Fig. 8b). All proteins were purified using fast protein liquid chromatography, coupled to Ni^2+^-NTA column (Qiagen), buffer-exchanged and further purified by Mono-Q anion-exchange chromatography (GE Biosciences). For Apaf-1 NOD protein, His_6_-tagged TEV protease was used to remove MBP from MBP-(His_6_)↓-NOD, and pure NOD was obtained by negative selection using Ni^2+^-NTA beads. Apaf-1 CARD-LZ-Avi (1 mg) was incubated with BirA (1 μg) in biotinylation buffer (50 mM bicine, pH 8.3, 10 mM ATP, 10 mM Mg(OAc)_2_ and 50 mM D-biotin (Sigma)) in a final volume of 1 ml for 30 min at 30 °C. The protein mixture was then dialysed into PBS to eliminate free biotin, and biotinylated Apaf-1-CARD-LZ-Avi was purified using an Avidin column and elution buffer containing D-biotin (2 mM) (Pierce Monomeric Avidin Kit; Thermo Scientific). The concentrations of all proteins were determined by the Bradford assay.

### SPR analysis

Association and dissociation of recombinant C9 proteins with Apaf-1-CARD-LZ-Avi was assessed by SPR analysis using a Biacore x100 biosensor (Biacore AB). Biotinylated Apaf1-CARD in SPR buffer (0.01 M HEPES, 0.15 M NaCl, 3 mM EDTA and 0.05% v/v Surfactant P20) was immobilized onto an SA sensor chip, according to the manufacturer's instructions. Recombinant C9 variants were buffer-exchanged into SPR buffer, and serially-diluted twofold, starting from 2 μM to 125 nM. Analyte solutions were passed over the surface of biosensor chips, beginning with the lowest concentration. Each measurement was performed at room temperature using a flow rate of 30 μl min^−1^ with a contact time of 120 s and a dissociation time of 700 s. The chip surface was regenerated after each measurement using the same buffer containing 1 M NaCl. Binding affinities were determined by equilibrium analysis, and kinetic data were fitted to the two-state binding model^40^,^41^.

### Gel filtration and multi-angle light scattering

Recombinant C9 proteins (200 μl; 40 μM) were injected onto a size-exclusion chromatography column (Wyatt Technology; catalogue number WTC030S5), using an AKTA purifier, and eluted in gel filtration buffer (20 mM HEPES, 50 mM NaCl, pH 8.0) at a flow rate of 0.5 ml min^−1^. Separated proteins/protein complexes were analysed using an in-line multi-angle light scattering detector (DAWN-HELEOS II, Wyatt Technology) and differential refractometer (Optilab T-rex, Wyatt Technology). Data were collected and molecular masses determined using the ASTRA software^42^. In other experiments, fully reconstituted Apaf-1-caspase-9 apoptosome complexes (see below) were fractionated using a Superose-6 gel filtration column^14^, and the proteins in each fraction (1 ml) were precipitated with trichloroacetic acid, separated by SDS–PAGE and western blotted using anti-Apaf-1 and C9 antibodies.

Similarly, Apaf-1 NOD complexes were assembled by incubating Apaf-1 NOD protein (1.5 μM) and ΔPro-C9-TM (80 nM) with or without dATP/MgCl_2_ (2 mM each) in a final volume of 200 μl at 37 °C for 30 min. The samples were then fractionated (1 ml fractions) by Superose-6 gel filtration chromatography (GE Healthcare) in gel filtration buffer with dithiothreitol (DTT; 20 mM HEPES, 50 mM NaCl 10 mM DTT, pH 8.0). The fractions were trichloroacetic acid precipitated, separated by SDS–PAGE and immunoblotted using an anti-T7 antibody. In related experiments, Apaf-1 NOD complexes were assembled in the absence of C9, fractionated and a portion of each fraction (23 μl) was incubated with ΔPro-C9-TM (200 nM) and ProC3* (500 nM) for 30 min at 37 °C. The reaction mixtures were then separated by SDS–PAGE and immunoblotted using anti-C9 and C3 antibodies.

### Apaf-1 apoptosome reconstitution and C9 activity assays

For most apoptosome reconstitution assays, full-length Apaf-1 (25 nM–2 μM), Cc (10 μM) and dATP/MgCl_2_ (2 mM each) were mixed with various recombinant C9 proteins (12.5–200 nM) and ProC3* (500 nM) in a final volume of 25 μl, and cleavage of ProC3* was used to assess C9 activity. In other assays, full-length and ΔPro-C9 proteins (25 nM–2 μM) were incubated with ProC3* (500 nM) in caspase activity buffer^16^ and C9 proteins were activated through the addition of ΔPro-C9* or Apaf-1 NOD protein (0.1–1.5 μM)±ammonium citrate (1 M). All samples were incubated at 37 °C for 10–60 min (as indicated in the figure legends), separated by SDS–PAGE and immunoblotted with anti-T7, Flag, C9 and/or C3 antibodies. All uncropped blots are provided in Supplementary Figs 9 and 10. Native lysates from *C9*^*−/−*^ mouse embryonic fibroblasts (10 mg ml^−1^) were used for some apoptosome reconstitution assays; following addition of recombinant C9 proteins, lysates were activated with Cc (10 μM) and dATP/MgCl_2_ (2 mM each) and incubated for 30 min at 37 °C. Finally, in other reconstitution assays, C9 LEHDase activity was measured on a SpectraMax M5 plate reader (Molecular Devices) using the fluorescent substrate LEHD.amc (500 μM), or through the use of a coupled DEVDase assay that measured the ability of C9 to activate ProC3, as previously described^14^.

### Incorporation of L-DOPA and site-specific crosslinking

Using an engineered *M. jannaschii* tRNA^Tyr^/tyrosyl-tRNA synthetase pair, L-DOPA was incorporated into the primary amino acid sequence of ProC9 in response to an artificially inserted amber stop codon (TAG), individually at each position in the GCFNF dimerization motif. Briefly, in a two-step transformation, wild-type and mutant ProC9 plasmids were first transformed into *E. coli* strain BL21(DE3)pLysS, followed by the PAC/tRNA-RS plasmid. Doubly transformed cells were then grown in amber flasks in M9-based glucose minimal medium (20 mM glucose, 2 mM MgSO_4_, 100 μM CaCl_2_, 3.37 mM Na_2_HPO_4_, 2.2 mM KH_2_PO_4_, 855 μM NaCl and 935 μM NH_4_Cl) to an optical density of 0.6–1.0, and protein expression was induced in the presence of L-DOPA (1 mM) and Na-ascorbate (4 mM) overnight at 16–18 °C. Cells were then lysed and the His_6_-tagged proteins were purified in the dark using Ni^2+^-NTA agarose in buffer containing DTT (1 mM). Importantly, only C9 proteins that incorporated L-DOPA were capable of translating the C-terminal His6 tag and thus only L-DOPA proteins could be purified on Ni^2+^-NTA beads. Furthermore, active L-DOPA-incorporated proteins were subjected to SDS–PAGE, transferred to nitrocellulose and stained using the nitroblue tetrazolium-Glycinate assay^28^. Apoptosome complexes were then reconstituted using recombinant Apaf-1 (300 or 1,500 nM), Cc/dATP, and ProC9-TM-F406DOPA or ProC9-TM-F404D/F406DOPA (20–200 nM). L-DOPA oxidation and site-specific crosslinking was triggered by the addition of sodium periodate (NaIO_4_, 1 mM) on ice for 1 h. In some cases, C9-TM-F406DOPA was also pulled-down using anti-T7-conjugated agarose beads (Novagen), in the presence of DTT (10 mM), separated by SDS–PAGE and immunoblotted using an anti-Apaf-1 antibody or a T7 antibody for C9.

### Data availability

All data generated or analysed during this study are included in this published article and its Supplementary Information Files.

## Additional information

**How to cite this article:** Wu, C.-C. *et al*. The Apaf-1 apoptosome induces formation of caspase-9 homo- and heterodimers with distinct activities. *Nat. Commun.* **7,** 13565 doi: 10.1038/ncomms13565 (2016).

**Publisher's note**: Springer Nature remains neutral with regard to jurisdictional claims in published maps and institutional affiliations.

## Supplementary Material

Supplementary Information Supplementary Figures 1 - 10 and Supplementary Table 1
