# Supplementary material for: The Apaf-1 apoptosome induces formation of caspase-9 homo- and heterodimers with distinct activities
Source: Nat Commun. 2016 Nov 24;7:13565. doi: 10.1038/ncomms13565 (PMC5123071; doi:10.1038/ncomms13565)
Supplement: Supplementary Information — Supplementary Figures 1 - 10 and Supplementary Table 1 [file ncomms13565-s1.pdf]

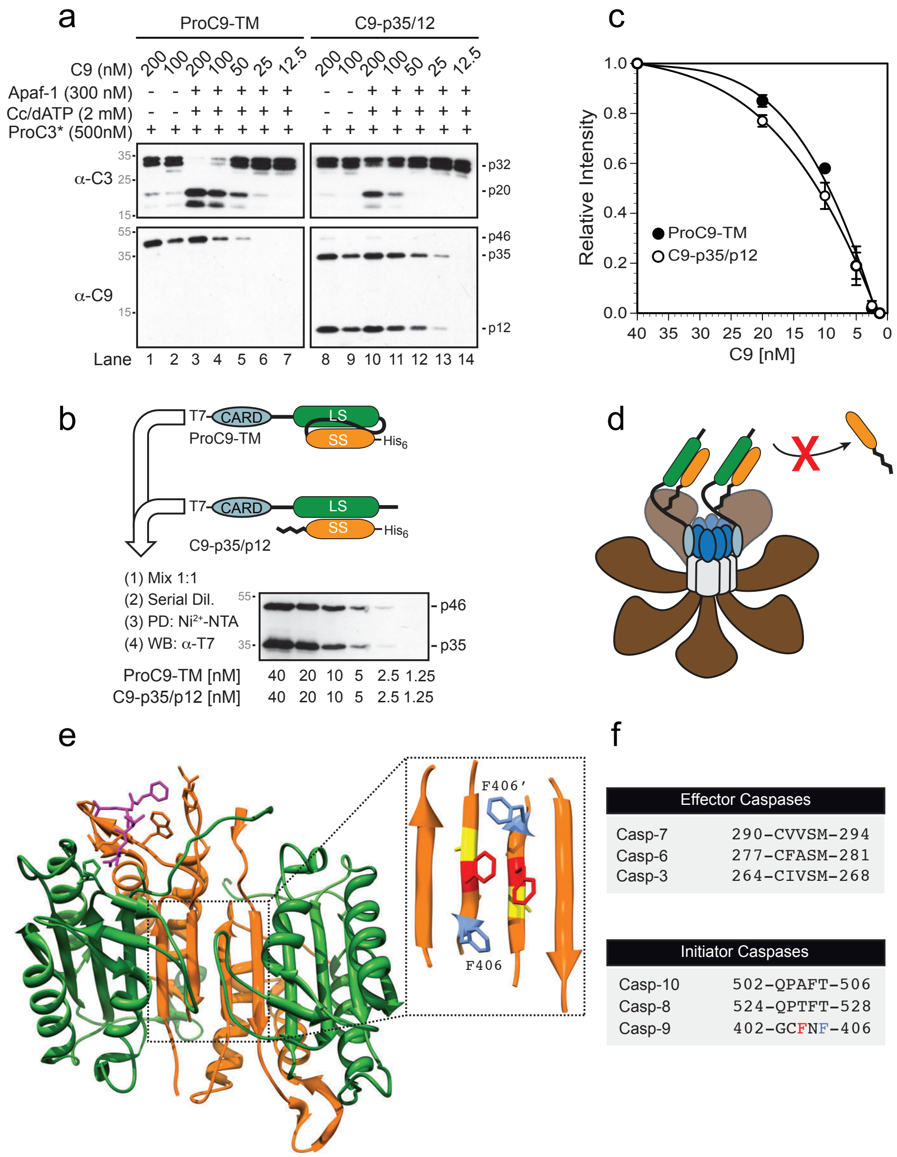

Supplementary Figure 1 | Reduced activity of C9-p35/p12, compared to ProC9, is not due to separation of its large and small subunits. (**a**) Apoptosome-bound ProC9-TM (12.5-200 nM) cleaves more ProC3* (500 nM) than does processed C9-p35/p12, particularly at low enzyme concentrations. (**b-d**) Recombinant ProC9-TM and C9-p35/p12 were mixed in equal parts (40 nM final concentrations for each). A serial dilution was then performed, followed by a pull-down of all C9 proteins *via* their C-terminal His_6 tags using Ni^{2+}-NTA beads. Finally, precipitates were immunoblotted for

N-terminal T7 tags, quantified by densitometry, and plotted against the starting C9 concentrations. Even at very low enzyme concentrations, the large and small subunits of C9-p35/p12 remained bound to one another with the p35 fragment being present at levels identical to those of full-length ProC9-TM. Thus, autoprocessing at Asp-315 does not initiate the release of C9-p35/p12 from the apoptosome through dissociation of its small p12 subunit from its large subunit. All experiments were repeated 3-5 times with similar results. (**e,f**) Crystal structure of homodimerized C9 (PDB: 1JXQ) with one active site bound to VAD.fmk (purple); the inset highlights the proposed GCFNF dimer interface, which binds in an anti-parallel fashion (C403, yellow; F404, red; F406, blue). Similar motifs mediate constitutive dimerization in C3, C6, and C7; whereas the dimer interface for C9 and C8/C10 differ substantially.

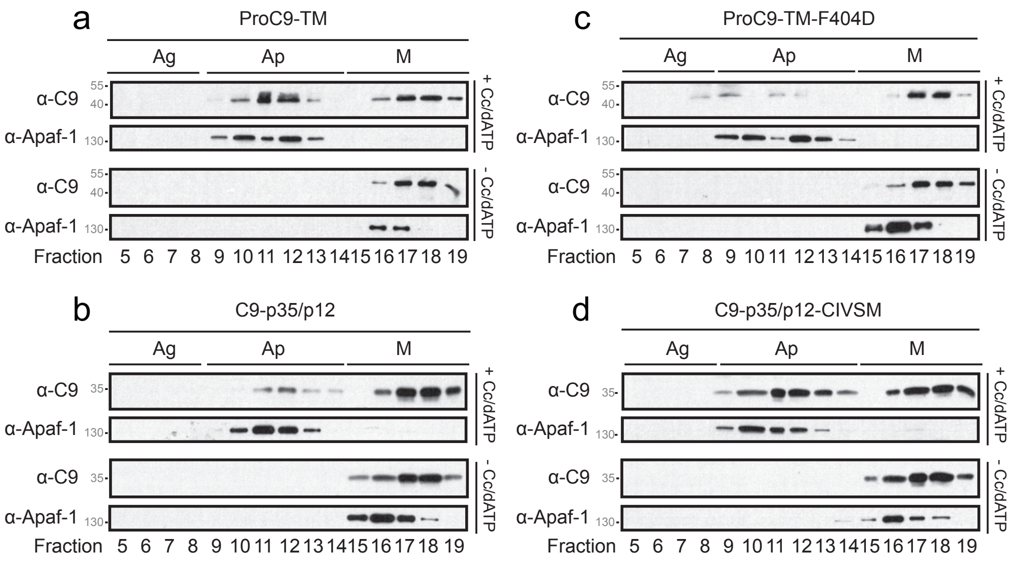

Supplementary Figure 2 | Recruitment of C9 proteins to the Apaf-1 apoptosome. (**a**-**d**) Apaf-1 (300 nM) was incubated with ProC9-TM, C9-p35/12, ProC9-TM-F404D, or C9-p35/p12-CIVSM (200 nM) ± Cc/dATP (2 mM each) for 30 min at 37ºC in a final volume of 200 μL. Following fractionation by Superose-6 gel filtration chromatography, the proteins in each fraction were precipitated with TCA and the resulting pellets immunoblotted for Apaf-1 and C9 proteins. The experiments were repeated three times with similar results.

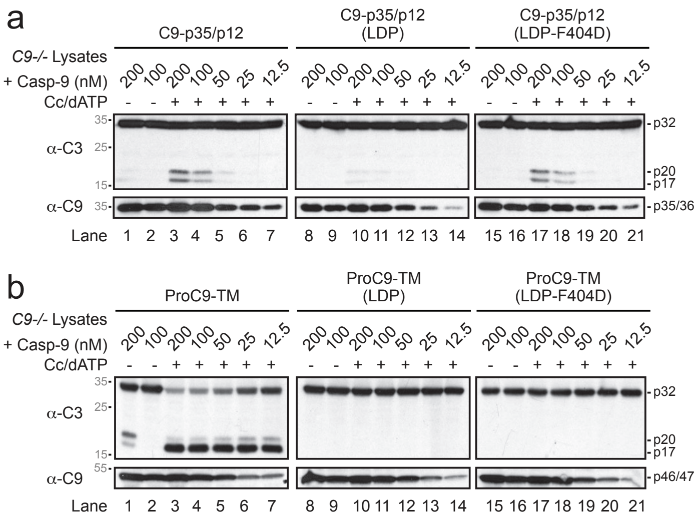

Supplementary Figure 3 | Reconstitution of *C9-/-* lysates with pro- and processed C9 proteins restores apoptosome function, but not when fused to dimer competent LDPs. (**a,b**) Lysates, prepared from *C9-/-* mouse embryonic fibroblasts, were reconstituted with increasing concentrations (12.5-200 nM) of recombinant C9-p35/p12, C9-p35/p12 (LDP), C9-p35/p12 (LDP-F404D), ProC9-TM, ProC9-TM (LDP), or ProC9-TM (LDP-F404D), activated with Cc (10 μM) and dATP/$MgCl_2$ (2 mM each), and assayed for cleavage of endogenous mouse ProC3. All experiments were repeated three times with similar results.

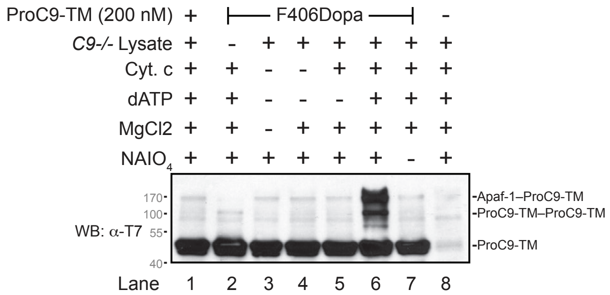

Supplementary Figure 4 | Cross-linkable ProC9-TM forms both homodimers and Apaf-1 heterodimers in Cc/dATP-activated *C9-/-* lysates. Lysates, prepared from *C9-/-* mouse embryonic fibroblasts, were reconstituted with ProC9-TM-F406DOPA (200 nM) and activated with Cc (10 μM) and dATP/$MgCl_2$ (2 mM each). The incorporated L-DOPA was then oxidized with sodium periodate, allowing the *ortho*-quinone in ProC9-TM-F406DOPA to react with C9 binding partners. Site-specific cross-linking of ProC9 with itself and endogenous Apaf-1 occurred only following assembly of the apoptosome.

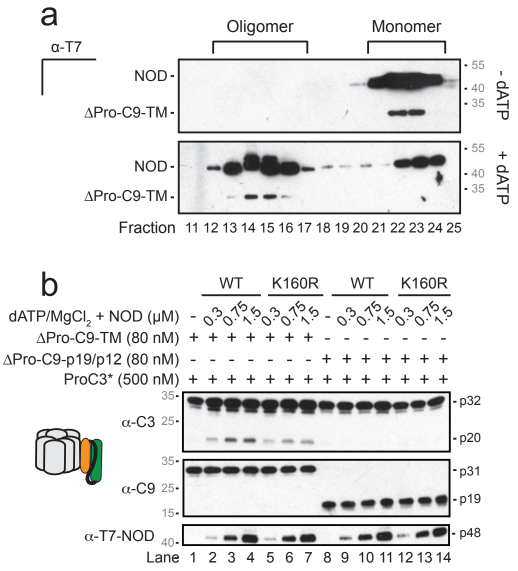

Supplementary Figure 5 | The Apaf-1 NOD binds directly to ProC9 and stimulates cleavage of ProC3. (**a**) T7-tagged Apaf-1 NOD protein (1.5 µM) and ProC9-TM (80 nM) were incubated ± dATP/$MgCl_2$ (2 mM each) in a final volume of 200 µL for 30 min at 37ºC. Following fractionation by Superose-6 gel filtration chromatography, the proteins in each fraction were precipitated with TCA and the resulting pellets immunoblotted for Apaf-1 NOD and ΔPro-C9-TM. (**b**) ΔPro-C9-TM and ΔPro-C9-p19/p12 (80 nM) were reconstituted with increasing concentrations of dATP/$MgCl_2$-activated Apaf-1 NOD, or the p-loop K160R mutant (0.3-1.5 µM), along with ProC3* (500 nM), and incubated for 30 min at 37ºC. Samples were then immunoblotted for Apaf-1 NOD, C9 and C3. All experiments were repeated three times with similar results.

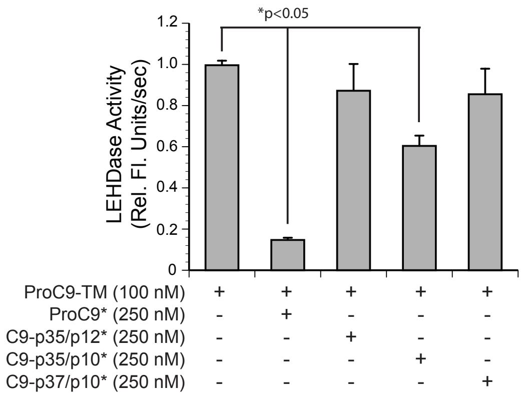

Supplementary Figure 6 | C9-p35/p10 possesses higher affinity for the apoptosome than either C9-p35/p12 or C9-p37/p10. ProC9*, C9-p35/p12*, C9-p35/p10*, and C9-p37/p10* proteins were generated as described in the methods. These catalytically-inactive pro- and processed C9 proteins (250 nM) were examined for their abilities to function as dominant-negative inhibitors and suppress apoptosome-dependent activation of active ProC9-TM (100 nM). ProC9* effectively inhibited apoptosome activation, as previously shown[14], whereas given their comparatively low affinities for the apoptosome, C9-p35/p12* and C9-p37/p10* failed to inhibit apoptosome activity. By contrast, C9-p35/p10* partially inhibited apoptosome activity, confirming that C9-p35/p10 possessed higher affinity for the apoptosome, compared to the other processed forms of C9. The experiment was repeated at least three times, and each bar represents the mean ± SEM. *$p<0.05$, ANOVA, Student-Newman-Keuls posthoc analysis.

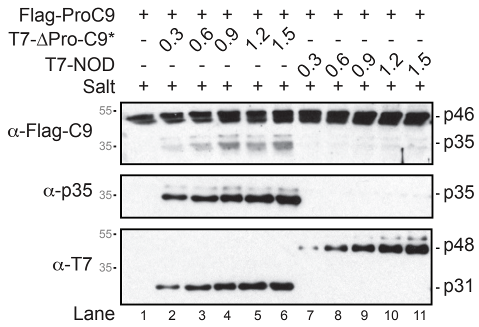

Supplementary Figure 7 | Ammonium citrate stimulates autocatalytic cleavage of ProC9 at Asp-315 within ProC9 homodimers but not within Apaf-1 NOD-ProC9 heterodimers. Wild-type ProC9 (80 nM) was incubated with increasing concentrations of either T7-ΔPro-C9* or T7-NOD (0.3-1.5 μM) for 30 min at 37ºC in the presence of ammonium citrate (1 M). Autocatalytic processing of ProC9 at Asp-315 was then determined by immunoblotting with an anti-Flag antibody and a p35-specific C9 antibody. Anti-T7 antibody confirmed equivalent concentrations of ΔPro-C9* and NOD proteins. The experiment was repeated three times with similar results.

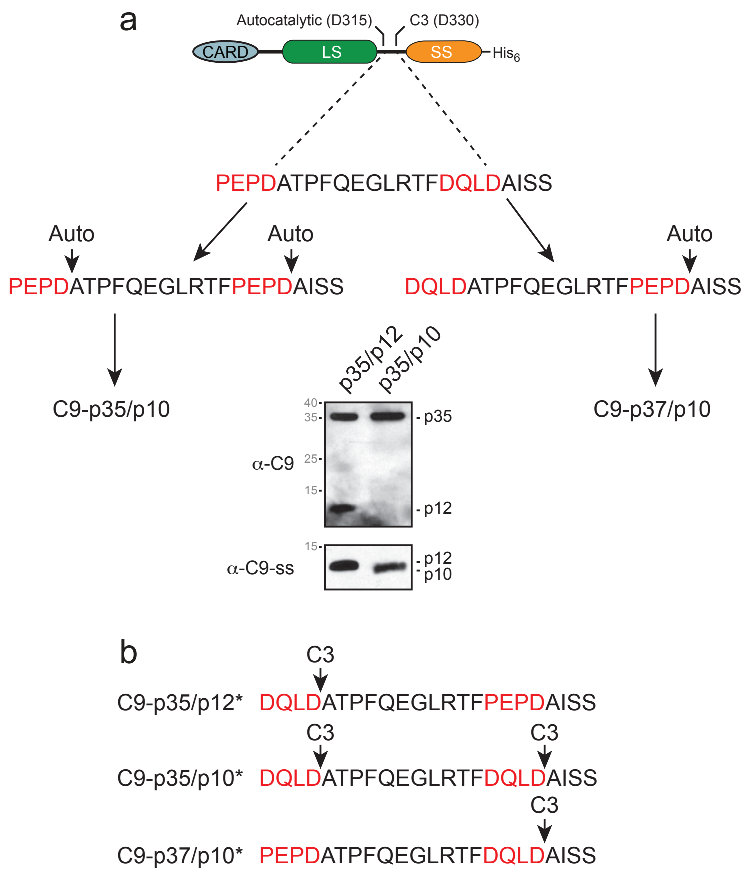

Supplementary Figure 8 | Generation of catalytically-active and inactive forms of processed C9. (**a**) ProC9 undergoes autoprocessing at PEPD^{315}↓A to generate the two-chain C9-p35/p12 enzyme, and both ProC9 and C9-p35/p12 can be cleaved by active C3 at DQLD^{330}↓A to generate C9-p37/p10 and C9-p35/p10, respectively. To generate recombinant C9-p35/p12, wild-type ProC9 was simply overexpressed in bacteria. Wild-type C9-p35/p10 was produced by substituting the DQLD sequence with PEPD, so that the entire linker could be autocatalytically removed during overexpression (left flow chart). Similarly, to generate recombinant C9-p37/p10, the PEPD and DQLD motifs were swapped for one another, so that cleavage would only occur at PEPD^{330}↓A (right flow chart). Recombinant C9-p35/p12 and C9-p35/p10 proteins were immunoblotted,

either with an antibody raised to the intersubunit linker, which recognizes both full-length C9 and its p12 subunit, or with an antibody raised to the C9 small subunit. (**b**) Using a similar approach as above, catalytically-inactive (C287A) forms of pro- and processed C9, as well as dimer mutants (C287A/F404D), were generated by incubating catalytically-inactive C9 mutants (described in A) with untagged active C3, which selectively cleaves C9 at DQLD↓A. See the methods for further details.

Fig. 1d,e

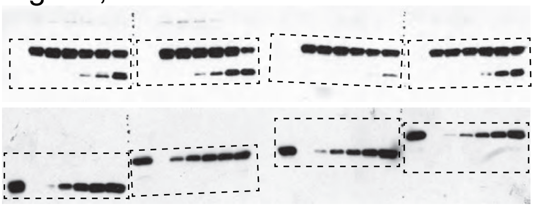

Fig. 4d

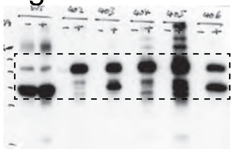

Fig. 4e

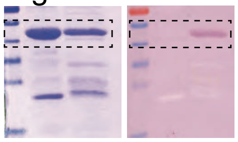

Fig. 1f,g

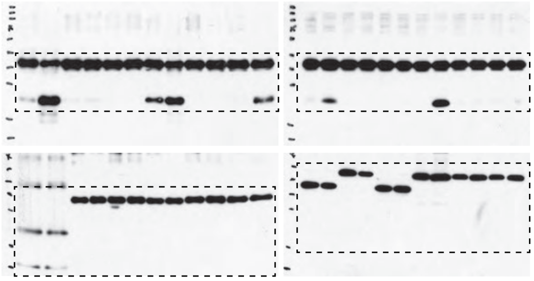

Fig. 4f (IP)

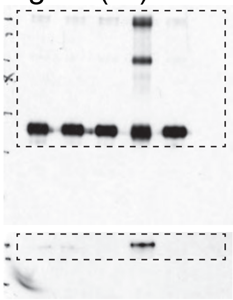

Fig. 4f (Input)

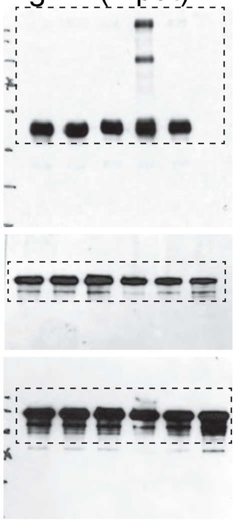

Fig. 2a

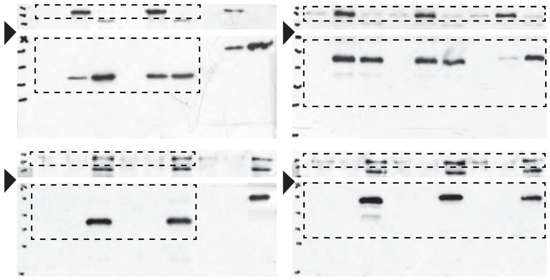

Fig. 4g

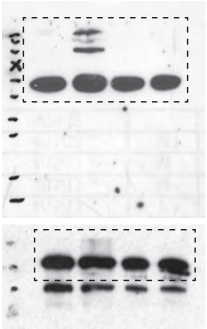

▶ indicates that the membrane was cut and immunoblotted with different primary antibodies.

Fig. 3b

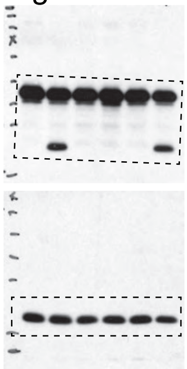

Fig. 3c

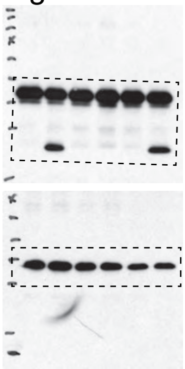

Fig. 3d

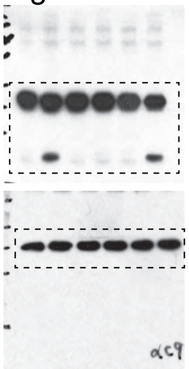

Fig. 3e

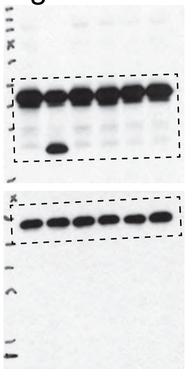

Fig. 3f

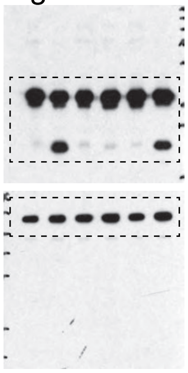

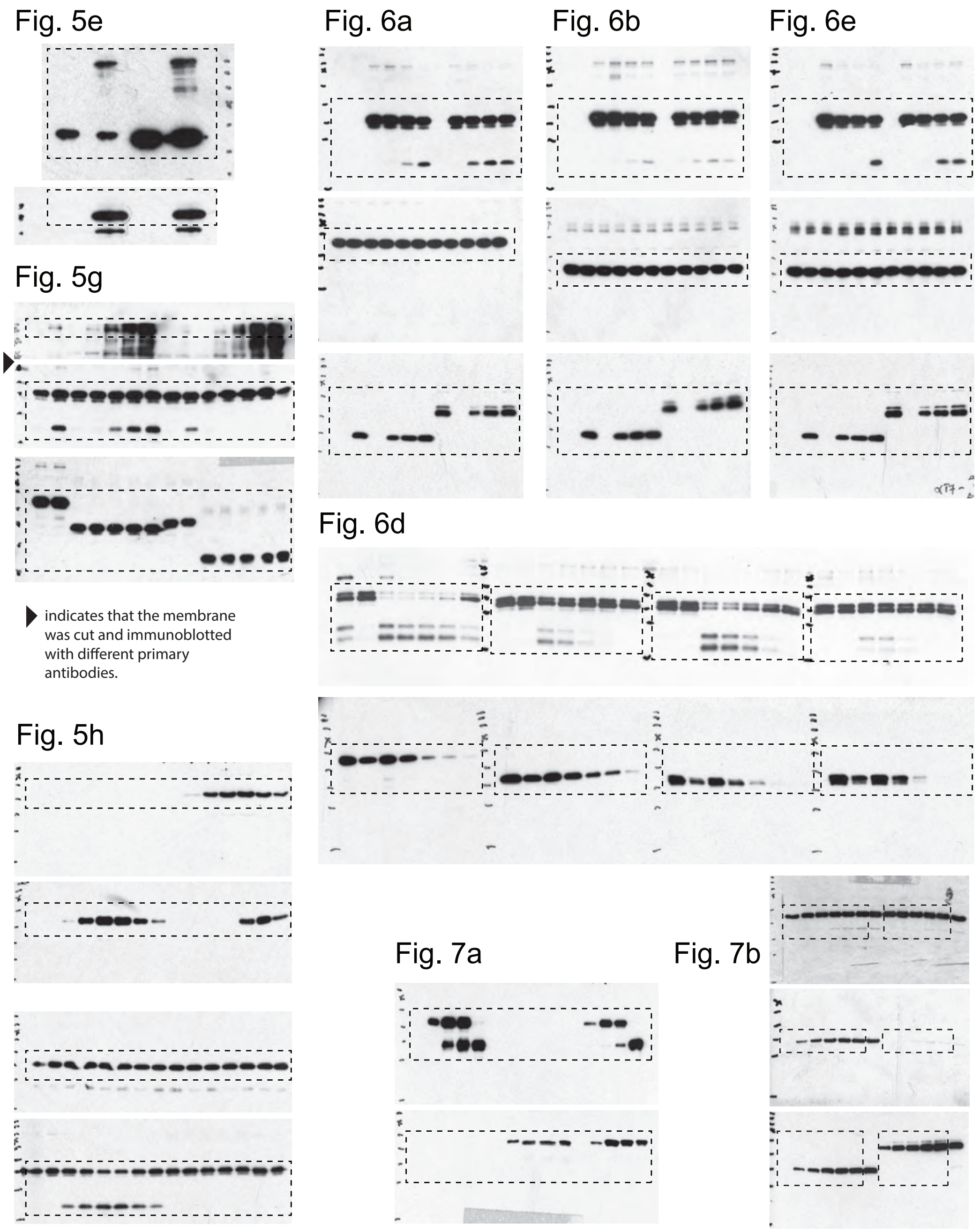

Supplementary Figure 9 | Uncropped images for main figures.

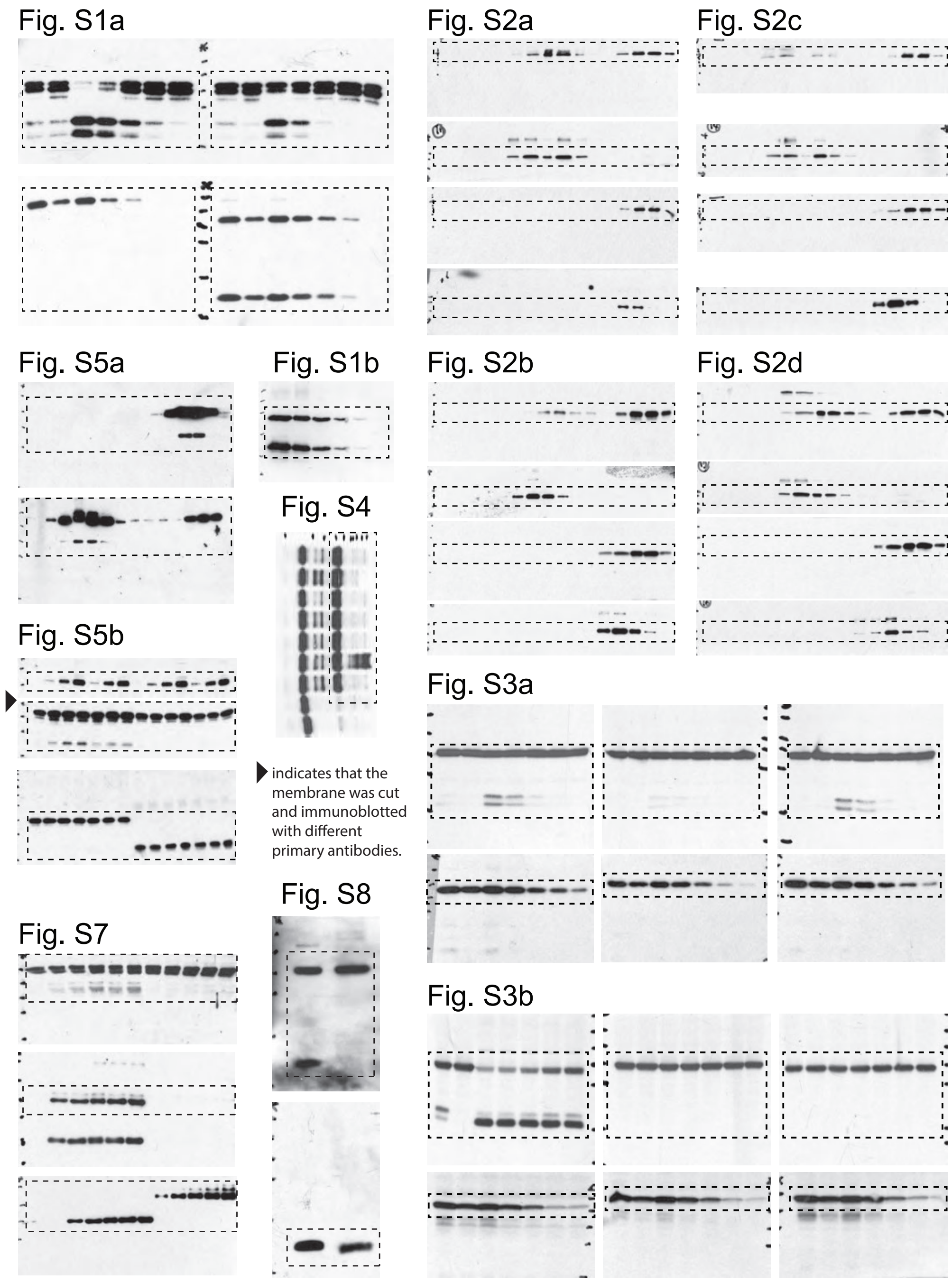

Supplementary Figure 10 | Uncropped images for supplementary figures.

Supplementary Table 1

Caspase Constructs	Mutations
Proenzymes	
ProC9	None
ProC9*	C287A
ProC9-F404D	F404D
ProC9*-F404D	C287A/F404D
ProC9-TM	E306A/D315A/D330A
ProC9-TM-F404D	E306A/D315A/D330A/F404D
ProC9-TM-CIVSM	ProC9-TM + G402C/C403I/F404V/N405S/F406M
ProC3	None
ProC3*	C163A
C9 prodomain and ∆Pro-C9 enzymes	
C9-CARD	None (aa 1-137)
∆Pro-C9*	C287A (aa 138-417)
∆Pro-C9-p19/p12	None (aa 138-417)
∆Pro-C9-p19/p12-F404D	F404D (aa 138-417)
∆Pro-C9-p19/p12-CIVSM	G402C/C403I/F404V/N405S/F406M (aa 138-417)
∆Pro-C9-TM	E306A/D315A/D330A (aa 138-417)
∆Pro-C9-TM-F404D	E306A/D315A/D330A/F404D (aa 138-417)
∆Pro-C9-TM-CIVSM	∆Pro-C9-TM + G402C/C403I/F404V/N405S/F406M
Active Two-chain Enzymes[§]	
C9-p35/p12	None
C9-p35/p12-CIVSM	G402C/C403I/F404V/N405S/F406M
C9-p35/p10	E306A/D327P/Q328E/L329P
C9-p37/p10	E306A/P312D/E313Q/P314L/D327P/Q328E/L329P
Inactive Two-chain Enzymes[§]	
C9-p35/p12*	C287A/P312D/E313Q /P314L/D327P/Q328E/L329P
C9-p35/p10*	C287A/P312D/E313Q/P314L
C9-p37/p10*	C287A
L-DOPA mutants	
ProC9-TM-F406Dopa	E306A/D315A/D330A/F406(amber stop codon)
ProC9-TM-F404D/F406Dopa	E306A/D315A/D330A/F404D/F406(amber stop codon)
Linker Dimer Peptides	
C9-p35/p12 (WT-LDP)	C9-p35/p12 with GGSGGSGCFNF fused to C-terminus
C9-p35/p12 (F404D-LDP)	C9-p35/p12 with GGSGGSGCDNF fused to C-terminus
ProC9-TM (WT-LDP)	ProC9-TM with GGSGGSGCFNF fused to C-terminus
ProC9-TM (F404D-LDP)	ProC9-TM with GGSGGSGCDNF fused to C-terminus
ProC9-TM (Scr-LDP)	ProC9-TM with GGSGGSFNGFC fused to C-terminus

[§]See Supplementary Fig. 8 schemes and the methods for the strategies utilized to generate catalytically-active and -inactive cleaved enzymes.
